# Three-Dimensional Imaging of Bioinspired Lipidic Mesophases Using Multicolored Light-Emitting Carbon Nanodots

**DOI:** 10.1021/acs.jpclett.4c00788

**Published:** 2024-06-11

**Authors:** Dominika Benkowska-Biernacka, Sebastian G. Mucha, Katarzyna Matczyszyn

**Affiliations:** † Institute of Advanced Materials, Faculty of Chemistry, Wroclaw University of Science and Technology, ul. Wybrzeze Wyspianskiego 27, 50-370 Wroclaw, Poland; ‡ Laboratoire Charles Coulomb (L2C), UMR5221, Université de Montpellier (CNRS), Campus Triolet, Place Eugene Bataillon, Montpellier 34095, France; § International Institute for Sustainability with Knotted Chiral Meta Matter (WPI-SKCM^2^), Hiroshima University, Higashihiroshima, Hiroshima 739-8526, Japan

## Abstract

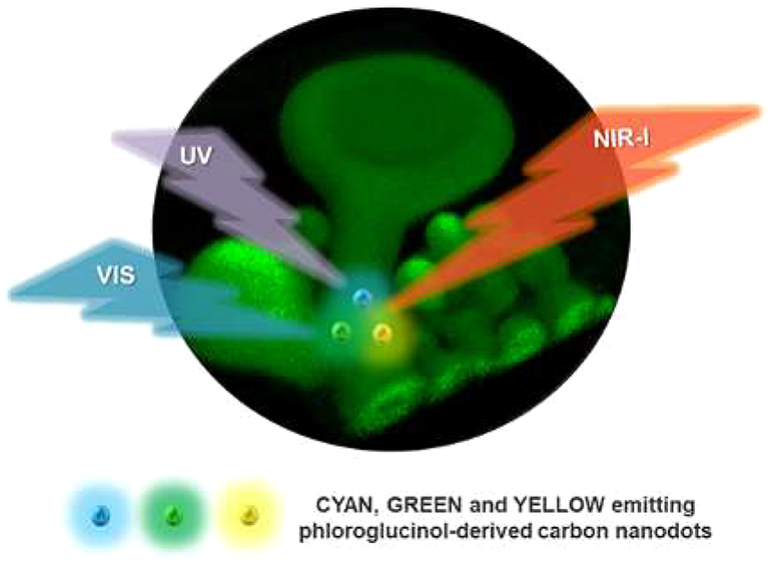

Recent progress in the design of carbon nanostructures exhibiting strong multiphoton-excited emission opens new pathways to explore the self-organization of lipids found in living organisms. Phospholipid-based lyotropic myelin figures (MFs) are promising materials as simplified models of biomembranes due to their structural resemblance to a multilamellar sheath insulating the axon. This study demonstrates the possibility of selective labeling of MFs by strongly emitting multicolor phloroglucinol-derived carbon nanodots (PG CNDs). Such dopants are efficiently excited by visible and near-infrared light; therefore, one- and two-photon fluorescence microscopies are incorporated to gain 3D insights into the MFs. Combining nondestructive fluorescence microscopy and spectroscopy techniques along with polarized light microscopy gives details on the stability and morphology of lipidic mesophases. Our findings suggest that PG CNDs can be a viable and simple alternative to conventional fluorescent lipid stains to image biologically relevant phospholipid-based structures.

Lipids are known to self-assemble into lyotropic mesophases, vital for biologically relevant entities.^1−4^ A prominent example of a lamellar liquid crystalline system of a biological origin is the myelin figure (MF).^5−7^ This structure resembles the lipid-rich myelin sheath, enabling the efficient conduction of nerve impulses in vertebrates.^8,9^ Typically, MFs are multilamellar cylindrical structures of self-organized concentrically wrapped layers of amphiphiles (e.g., lipids, as well as ionic and nonionic surfactants) alternating with layers of water.^10−15^ Such three-dimensional (3D) organization can spontaneously occur once surfactant molecules combine with aqueous media. A deeper comprehension of the architecture, stability, and response to external stimuli of lipidic liquid crystalline materials may lead to a better understanding of native structures and advances in the design of biomimetic systems.^4,16−20^

Since phospholipids constitute 40% of lipid composition in myelin sheath and the majority of phospholipids in mammalian cells are phosphatidylcholines (PC),^8,21^ assemblies based on PCs can serve as simple models of the biologically significant membranes.^22,23^ Research on lamellar structures made of commercially available PCs using high-resolution tools applied in the study of lyotropic liquid crystals may offer deep insights into the sample.^24^ Considering an incomplete understanding of the etiology behind pathological alterations in the native myelin, the application of noninvasive techniques (such as multiphoton fluorescence microscopy) can provide new ways to follow fine morphological changes within the lipidic mesophases in living systems and shed light on demyelinating diseases.^25,26^ Although studies on the use of the nanoparticles (NPs) interacting with simplified biological membranes have potential in diagnostic and therapeutic,^27,28^ the incorporation of nanomaterials into research on MFs has rarely been addressed in previous works. It was reported that metal oxide NPs can affect the growth and morphology of phospholipid-based MFs.^29^ Moreover, carbon nanodots (CNDs) have been successfully applied in staining the aqueous phase in multilamellar lipid tubes.^30^ However, there is still a lack of studies on doping hydrophobic regions of MFs with NPs and imaging lipidic mesophases with fluorescent probes offering multicolor emission upon excitation in biological windows.^31^ While using commercial organic dyes (*e.g*., Nile Red and BODIPY) for lipid labeling is conventional practice, exploring the use of nanomaterials in this context is compelling.^32−35^

Among luminescent NPs, CNDs are in line with the trend of environmentally friendly materials and sustainable processes.^36−40^ These carbogenic nanostructures can exhibit excellent photoluminescence performance, such as high-emission quantum yield (QY), optical stability, and narrow emission spectrum.^30,37,41−44^ Moreover, the unique optical properties of CNDs can be customized for specific applications through the selection of a proper precursor and precise control of the synthesis conditions.^45,46^ Despite extensive work in the field of coupling CNDs to biomimetic membranes, vast research has focused on studies of lipid-based structures in the form of vesicles.^38,47,48^ Here, we report the potential of phloroglucinol-derived carbon nanodots (PG CNDs) as fluorescence probes to conduct 3D optical imaging of PC-based elongated microstructures. We show the influence of PG CNDs on the thermal stability and formation of MFs composed of 1,2-dilauroyl-*sn*-glycero-3-phosphocholine (DLPC) and 1,2-dimyristoyl-*sn*-glycero-3-phosphocholine (DMPC). Using confocal fluorescence microscopy (CFM), we identified different morphologies of MFs, focusing on structures consisting of micrometer-diameter water cores. Besides, we characterize the emission properties of multicolored PG CNDs in a lipid matrix excited in the nonlinear regime. Then, we use two-photon microscopy to image lipidic mesophases, presenting a new concept of labeling the hydrophobic parts of MFs with CNDs.

The formation of MFs was observed upon introduction of water to dried lipid droplets using a polarized light microscope equipped with a heating stage. An essential factor in the growth of elongated multilamellar structures (composed of DLPC or DMPC) is the temperature at which PCs change their physical state from an ordered gel to a liquid crystalline state (*T*_m_). As shown in Figure 1a, using DMPC (*T*_m_ = 24 °C) example, the hydrated lipid reservoir can be incubated below *T*_m_ without forming MFs. Once the sample is heated to *T*_m_, the rod-like microstructures begin to grow from the lipid–water interface toward the aqueous phase (Figure 1b,c).

**Figure 1:**
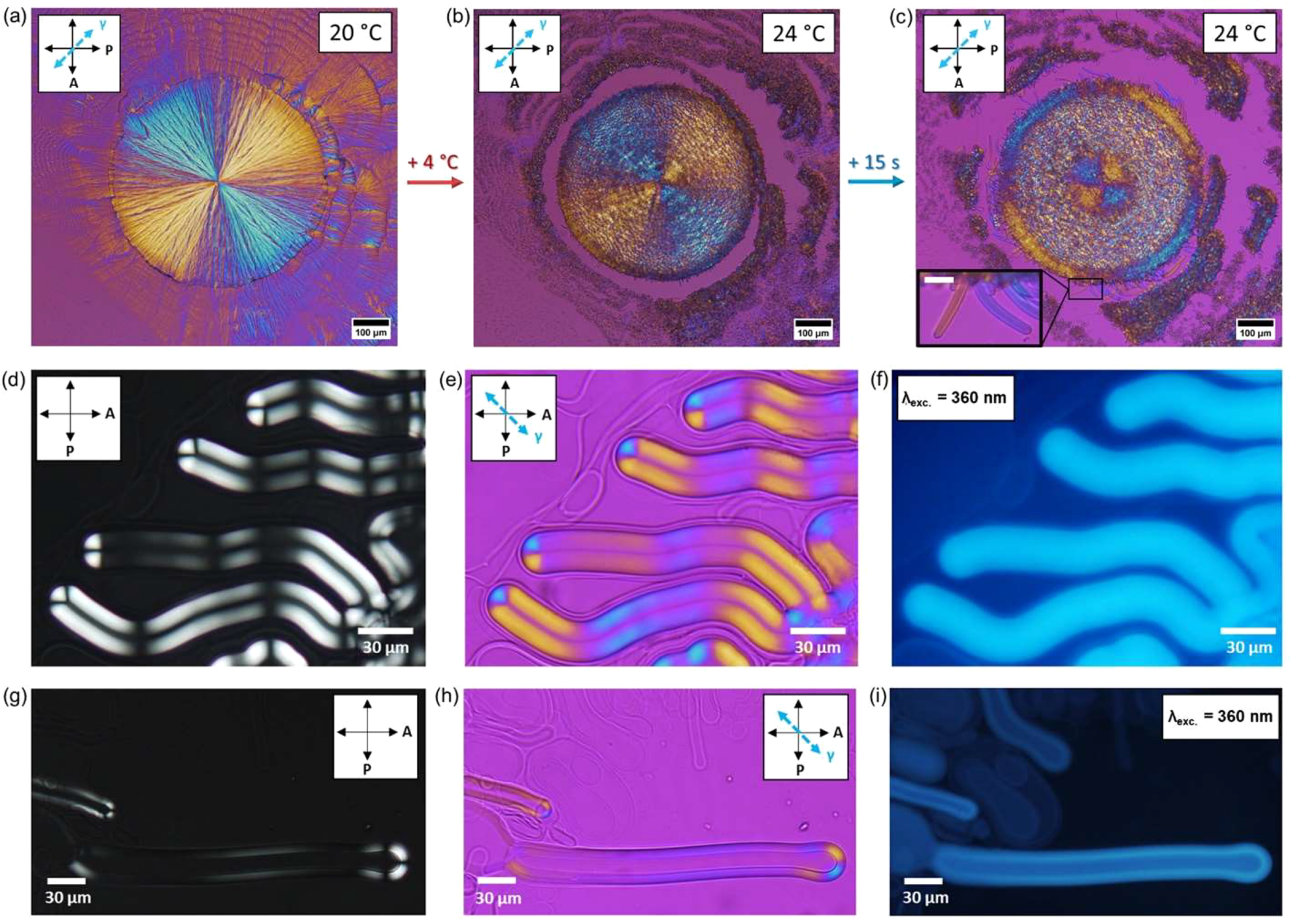
Polarized light images showing changes across the DMPC–water interface (a) at 20 °C, (b) at 24 °C, and (c) after 15 s at 24 °C. Micrographs were obtained with a full-wave retardation plate placed between crossed polarizers. The orientations of the polarizer (P) and analyzer (A) are indicated by black double-sided arrows, and the direction of the slow axis of the retardation plate (γ) is marked as a blue double arrow. Scale bars are (a–c) 100 and 20 μm (enlarged image from panel c). (d–i) DMPC-based MFs doped with CYAN CNDs formed (d–f) at the edge of the lipid droplet and (g–i) from the preformed multilamellar structures. The samples imaged between (d, g) crossed polarizers and (e, h) crossed polarizers with an additionally inserted full-wave retardation plate. (f, i) Fluorescence microscopy images of corresponding regions of the samples (λ_exc._ = 360 nm). Scale bars in panels d–i represent 30 μm.

Then, the samples of PCs-based MFs were marked with PG CNDs. Three types of PG CNDs in ethanol (EtOH) dispersions were selected for further investigations (i.e., CYAN, GREEN, and YELLOW CNDs). The detailed structural and optical characterizations of applied PG CNDs (including elaboration on fluorescence mechanisms) were presented in a previous paper.^49^ Based on high-resolution transmission electron microscopy analysis, PG CNDs have irregular shapes and average diameters of ∼4 nm (exemplary results are in Figure 2a). As illustrated in Figure 2b, investigated PG CNDs show lattice spacing of ∼0.32 nm (similar to the interlayer distance of 0.34 nm for the (002) plane in graphitic materials).^50^ Furthermore, PG CNDs consist of polar (e.g., hydroxyl groups) and nonpolar (methyl and methylene moieties) structural units. Their extensive structural characterization unravelled the predominant role of nonpolar carbogenic domains.^49^

**Figure 2:**
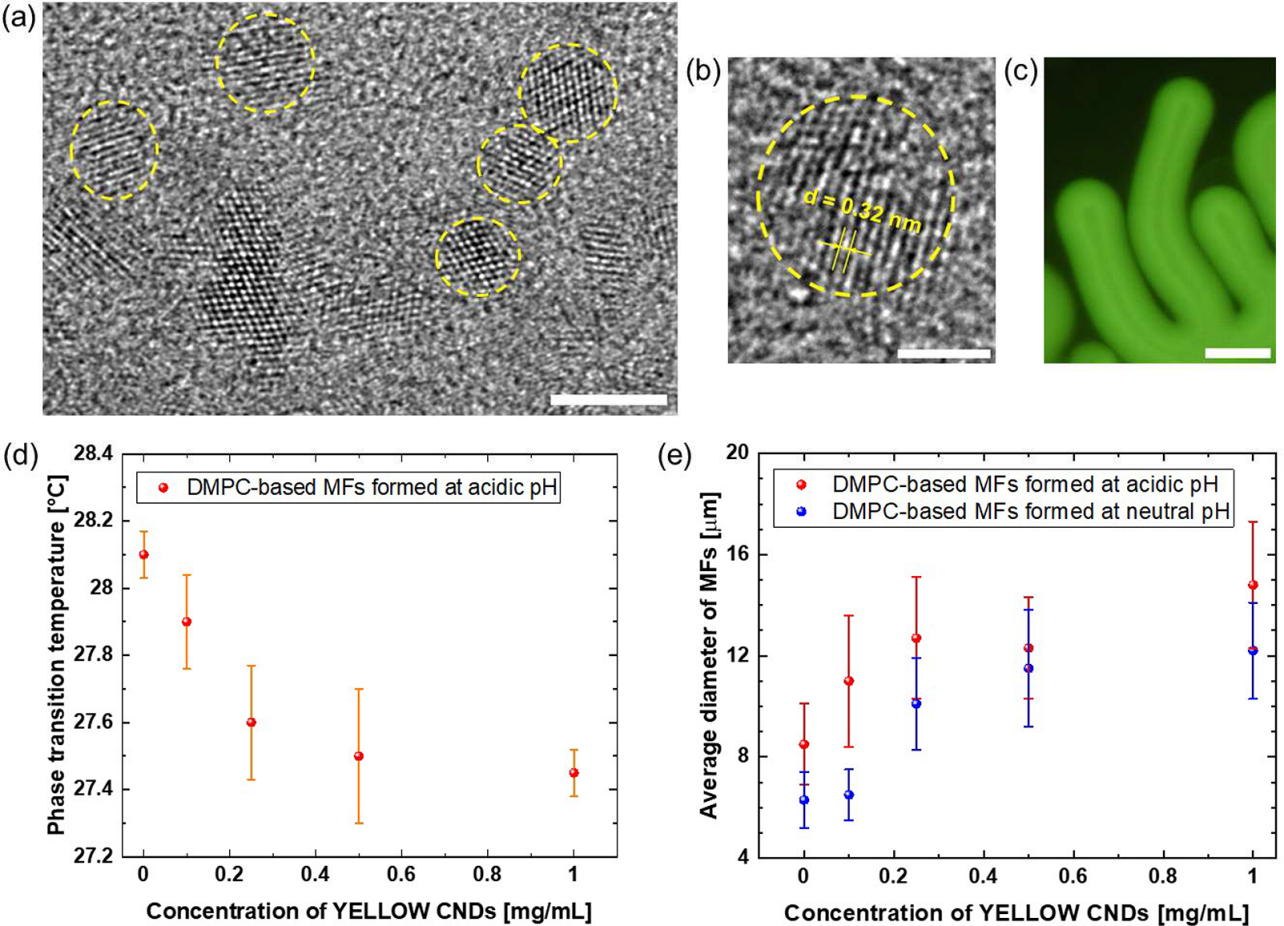
(a, b) TEM images of YELLOW CNDs. (c) Widefield fluorescence microscopy image of MFs with YELLOW CNDs (at initial nanoparticle concentration of 1 mg/mL) formed at acidic pH (λ_exc._: 460–495 nm). Scale bars are (a) 5 nm, (b) 2 nm, and (c) 20 μm. (d) Scatter plot indicating the temperature at which DMPC-based MFs start to form with and without YELLOW CNDs after hydration of a dried lipid droplet with a solution at pH 1. (e) Graph depicting the average diameter of DMPC-based MFs formed with and without YELLOW CNDs at acidic (red dots) and neutral (blue dots) pH.

Selected PG CNDs exhibit monochromatic and tunable emission colors in low-carbon alcohols, as evidenced by the narrow emission bands at 478 nm (CYAN CNDs), 509 nm (GREEN CNDs), and 550 nm (YELLOW CNDs) in Figure S1. PG CNDs are excellent fluorophores (e.g., the QY of CYAN CNDs is around 62% in methanol (MeOH) dispersion and 34% in Tris Buffer) and efficient two-photon absorbers (*e.g*. two-photon absorption merit factor reaches 0.31 GM·mol·g^–1^ at 740 nm for YELLOW CNDs; Table S1).^49^ Additionally, PG CNDs provide broadband two-photon absorption (CYAN CNDs: 700–950 nm; GREEN CNDs: 700–1000 nm; YELLOW CNDs: 750–1100 nm), which fully covers the first biological window (NIR-I: 700–950 nm).^31,49^ What is more, PG CNDs exhibit high purity of the emission color due to small full-width at half-maximum (FWHM; CYAN CNDs: ∼32 nm; GREEN CNDs: ∼33 nm; YELLOW CNDs: ∼78 nm in MeOH dispersions; Figure S1). Compared to the commercial lipophilic stain Nile Red (FWHM ∼62 nm; Figure S2a), CYAN and GREEN CNDs exhibit narrower emission spectra. In the case of YELLOW CNDs, the FWHMvalue is comparable with Nile Red’s value. Moreover, these yellow-emitting NPs have a significant Stokes shift (3186 cm^–1^), which may benefit multiplex imaging.^49,51,52^ Furthermore, PG CNDs show excellent photostability under prolonged illuminations. As illustrated by the example of YELLOW CNDs in comparison with Nile Red (Figure S2b), CNDs exposed to continuous NIR-irradiation with the pulsed laser beam (λ = 740 nm, *P*_laser_ = 10 mW) also maintain a high fluorescence intensity. Details of all of the experimental methods are given in the Experimental Section of the Supporting Information.

The possibility of obtaining the characteristic textures of MFs after doping the bilayers with PG CNDs was evaluated by using polarized light microscopy (PLM). Over time, distinctive multilayered structures were formed, taking straight, oval, and looped shapes in samples with different fractions of studied PG CNDs (Figures 1d and S3). Such various forms were also reported in purely lipidic MFs in the aqueous environment.^17,53^ The use of a full-wave plate introducing relative retardation for green light (530 nm) provided qualitative insight into the orientation order of the lipids across the MFs. The vivid interference colors show regions where the long axes of the PCs are oriented perpendicular (yellow) or parallel (blue) to the slow axis of the retardation plate. In addition, applying the full-wave plate improved the contrast and allowed for more detailed micrographs to be obtained under the same light source. Figure 1e shows that the characteristic organization of lipid molecules along the pristine MFs is also observed in mesophases doped with PG CNDs.^15,53^ The amphiphilic molecules are self-organized over the length of the MFs in such a way that they are arranged into a concentric lamellar structure.

Subsequently, we confirmed the presence of PG CNDs in lipidic mesophase by wide-field fluorescence microscopy. As shown in Figure 1f, the one-photon excited (OPE) emission along the lipid tubes stained with CYAN CNDs was intense and uniformly distributed within the structures that emerged from the PC/water interface (λ_exc._ = 360 nm). Also, there was no accumulation of nanostructures near the roots of the MFs. A comparison of polarized light images and fluorescence images shows that some thin MFs might only be evident by the second technique (Figure 1g,i). Using strongly emitting PG CNDs enabled efficient imaging of multilamellar forms with fewer bilayers or a high ratio of the diameter of the water core to the entire lipid tube. Based on the elongated structure shown in Figure 1i, it is apparent that there was a much lower emission intensity in its central part compared with the multilayered walls. This local drop in intensity can be explained by a PG CNDs-free volume running inside the MFs.

Prior to further investigating the distribution of dopants, we examined the influence of PG CNDs on the liquid crystalline properties of MFs. The studies were performed on structures marked with YELLOW CNDs (0.1–1.0 mg/mL), nanostructures with the highest contribution of oxygenous groups, and solvent-responsive fluorescence properties.^49^ Nearly homogeneous emission detected along walls of characteristic MFs formed with YELLOW CNDs (even with their initial concentrations of 1 mg/mL) indicated uniform NP distribution within the hydrophobic parts of investigated lipidic mesophases (Figure 2c). The first experiments were carried out on DMPC-based MFs after adding an aqueous phase at pH 1 and 7. According to Figure 2d, the phase transition temperature, at which lipid tube growth begins, decreases with increasing dopant concentrations for samples prepared at acidic pH. The strength and direction of association between the concentration of YELLOW CNDs and *T*_m_ were evaluated by Spearman’s rank correlation test. The statistically very strong negative correlation was confirmed (*r*_s_ = −0.8, *p*-value < 0.001, where *r*_s_ is Spearman’s rank correlation coefficient). In contrast, the data for DMPC-based MFs at neutral pH (Figure S4a) showed a statistically insignificant correlation between the concentration of dopants and *T*_m_ (*p*-value = 0.17).

The effect of PG CNDs on the diameters of lipid tubes was then investigated within samples composed of DMPC and its shorter acyl chain analogue DLPC (*T*_m_ = −2 °C). The diameters of the DMPC-based MFs were measured at acidic and neutral pH. In accordance with the earlier studies,^16^ larger diameter tubes were formed by lowering the initial pH of the aqueous phase. This relationship is attributed to the protonation of the phosphate group (p*K*_a_ < 2)^54^ within the lipid headgroup. As shown in image Figure 2e, such a trend is observed over the entire range of concentrations of PG CNDs. A very strong positive correlation between the diameter of MFs and the concentration of dopants was demonstrated for DMPC-based MFs at neutral pH (*r*_s_ = 0.8, *p*-value < 0.001), while moderate positive association DMPC-based MFs at acidic pH (*r*_s_ = 0.6, *p*-value < 0.001). In the case of DLPC-based MFs formed at neutral pH, a fair positive correlation was confirmed (*r*_s_ = 0.4, *p*-value <0.001; Figure S4b). Therefore, the addition of CNDs to lipid mesophases may result in a significant change in their dimensions, depending on the dopant concentration.

In order to characterize the 3D architecture of the MFs, we applied CFM. Experiments were performed on DLPC-based MFs doped with PG CND (0.1 mg/mL). As shown using the example of MFs with GREEN CNDs, an elongated internal nonfluorescent volume could be observed along the lipid tube (Figure S5a–d). According to previous findings for samples stained with commercial dyes (Texas Red and Nile Red),^53,55^ the distinct region inside the MFs is a lipid-free water core. These studies revealed a relationship between the adhesion of membranes to the substrate and the shape of the water core within the MFs. For instance, MFs growing from preformed multilayer structures, *i.e*., from a detached lipid reservoir region, show a “hollow” cross-sectional view. In contrast, MFs formed from the attached edge of the lipid droplet have a “solid” cross-sectional view.

The ability of PG CNDs to effectively mark diverse morphologies of MFs was also validated by confocal slides of oval-ended MFs (Figure 3). The cross section of DLPC-based MFs doped with CYAN CNDs (λ_exc._ = 405 nm, λ_em_: 440–480 nm) indicated that the microstructure was flat across the rounded tip. The following sample, marked with GREEN CNDs (λ_exc._ = 488 nm, λ_em_: 510–520 nm), showed a characteristic cross section of lamellar collapsed structures.^12,53^ The sample doped with YELLOW CNDs (λ_exc._ = 488 nm, λ_em_: 530–570 nm) exhibited a similar transverse view, lipid-free inner volume, and prominent fluorescent boundaries. The thickness of the stacked lipid bilayers along the entire length of the structure remains constant, although the width of the front part of the tube might increase because of the fluidity of the lamellar mesophase. Each of the 3D scans confirmed the homogeneous distribution of PG CNDs in the lipid bilayers surrounding the water core. There was no difference in the emission intensity along areas of the lamellar mesophase wrapped around the nonfluorescent channel. Additionally, the confocal z-stacks of straight and oval-ended MFs were 3D reconstructed and provided morphological details of internal and external structures. Illustrative projections of oval-ended forms demonstrated a cylindrical shape over the long axis of the MF and a pronounced concavity of the rounded tip (Figure S5e).

**Figure 3:**
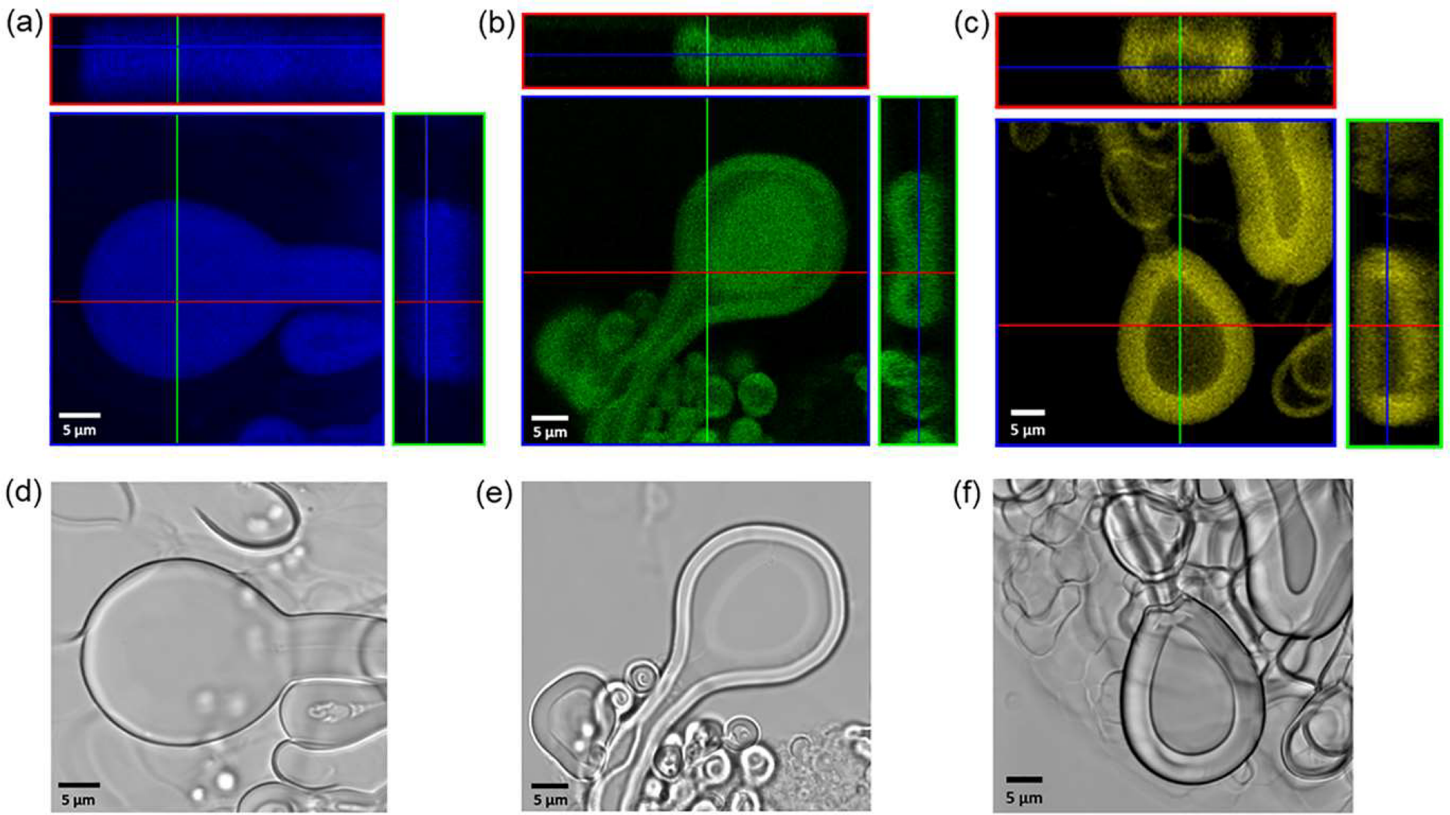
Fluorescence confocal images of MFs doped with (a) CYAN, (b) GREEN, and (c) YELLOW CNDs taken upon excitation at (a) 405 nm and (b, c) 480 nm. The emission signal was collected at wavelength ranges (a) 440–480 nm, (b) 510–520 nm, and (c) 530–570 nm. Colored frames of the images (red, green, and blue) are related to the colors of the lines indicating spatial locations at which the cross sections of all views were taken. (d–f) Corresponding bright field microscopy images. Scale bars are 5 μm.

Since CYAN CNDs were excited at a wavelength in the UV, we performed further imaging of nature-inspired structures using excitation wavelengths from the NIR-I region. For this purpose, we applied two-photon excited fluorescence microscopy (TPEFM), which overcomes many of the limitations of conventional (*i.e*. one-photon) techniques, allowing deeper penetration depth and better image contrast.^31,56^ Furthermore, incorporating multiphoton microscopy for lipidic mesophases aligns with the current trend of imaging nerve fibers in high-volume samples.^32,57^ As shown in Figure 4, samples doped with CYAN CNDs can be successfully excited at 850 nm in lipid tubes with diameters above 20 μm. PLM allowed a preliminary qualitative verification of the sample, *e.g.*, in terms of lipid arrangement (Figure 4a,b). The subsequent application of TPEFM revealed distinct parts of the multilayered microstructure where the presence or absence of emission signals could be detected. A scan at half the sample height showed an internal nonfluorescent region within MFs (Figure 4c), while an intensity map taken 4 μm above indicated that it is a confined volume (Figure 4d). These observations are consistent with the results mentioned above obtained by CFM.

**Figure 4:**
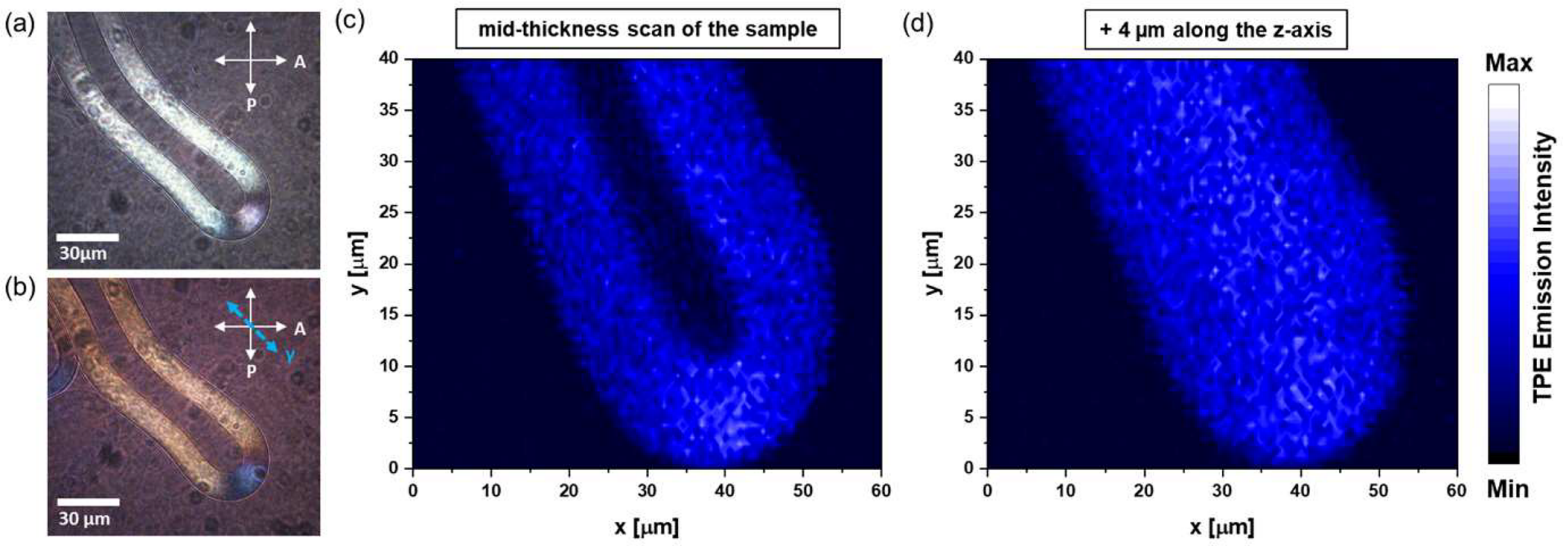
(a, b) DLPC-based MFs doped with CYAN CNDs imaged using PLM. White and blue arrows represent the orientation of crossed polarizers (P, A) and the slow axis of the full-wave retardation plate (γ). Scale bars are 30 μm. (c, d) TPE fluorescence intensity maps of the same DLPC-based MF taken at mid-height of the sample and 4 μm higher. Both scans are depicted using the same intensity scale. The sample was excited at 850 nm.

Then, we experimentally confirmed that PG CNDs in doped PC-based MFs exhibit multiphoton excited fluorescence. Verification of the character of the observed process was revealed by detecting the luminescence intensity at a series of low excitation powers (<3 mW) for all tested fractions of PG CNDs in the DLPC matrix. The linear dependencies with the slope of ∼2 for the double logarithmic plots of emission intensity vs average excitation laser power indicated the two-photon nature of the processes involved in all samples (Figure 5a), comparable with the slope values for pure CNDs in the MeOH.^49^ Next, the two-photon excited (TPE) fluorescence spectra of PG CNDs in the lipidic matrix were performed upon excitation in the NIR-I region. As shown in Figure 5b, the TPE maxima of CYAN, GREEN, and YELLOW CNDs in MFs peaked at 487, 536, and 566 nm, respectively.

**Figure 5:**
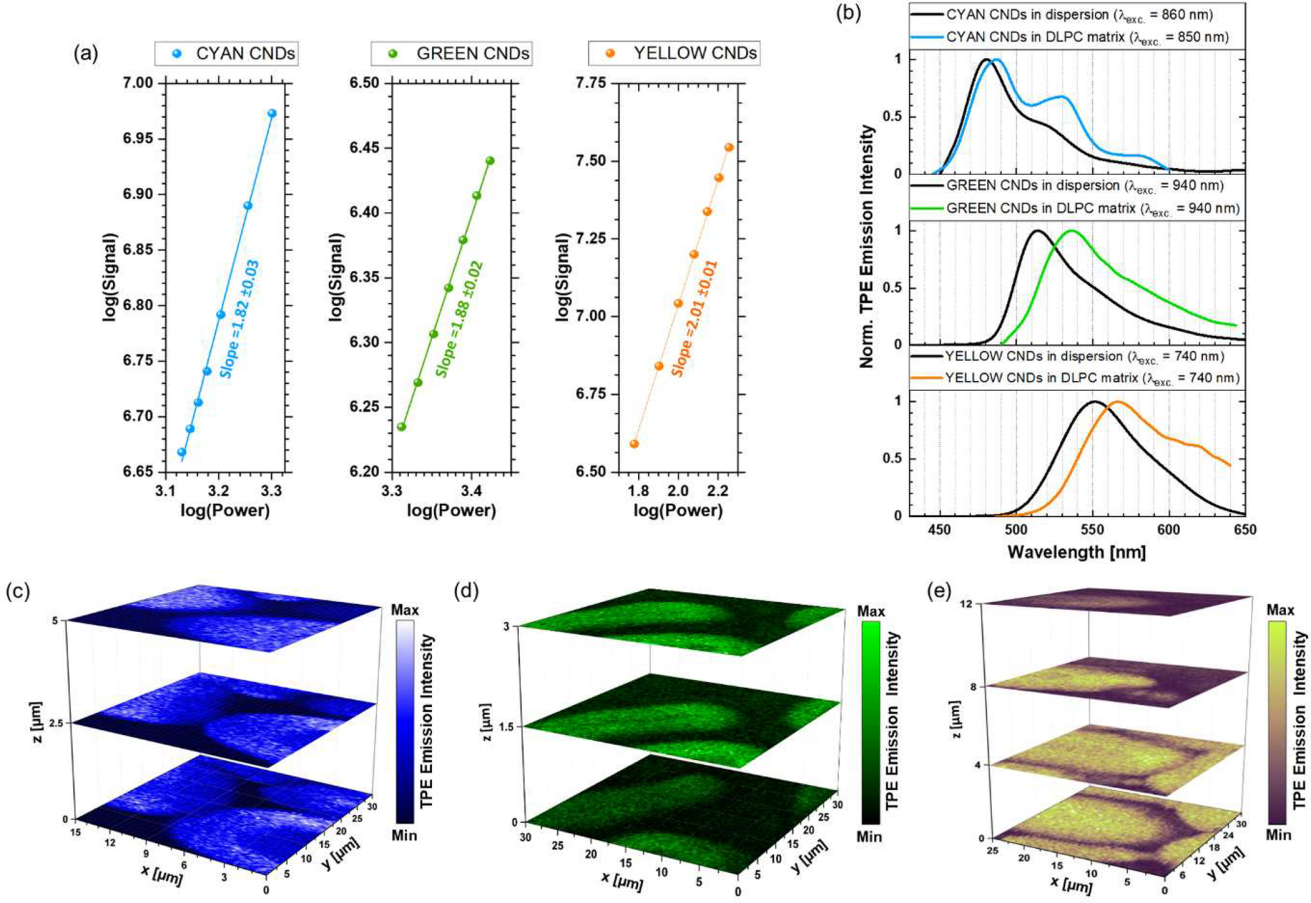
(a) Dependence of emission intensity of CYAN, GREEN, and YELLOW CNDs upon average excitation power. The lines represent linear fits of the data; the slopes are 1.82 ± 0.03 (CYAN CNDs), 1.88 ± 0.02 (GREEN CNDs), and 2.01 ± 0.01 (YELLOW CNDs). (b) TPE emission spectra of PG CNDs in alcohol dispersion (black lines) and lipid matrix (colored lines). (c–e) Stacks of TPE emission intensity raster scans of MFs doped with (c) CYAN, (d) GREEN, and (e) YELLOW CNDs. All CND-doped DLPC-based structures were excited at 850 nm (CYAN CNDs), 940 nm (GREEN CNDs), and 740 nm (YELLOW CNDs).

According to the results summarized in Table S2, all fractions of PG CNDs in lipidic lamellar mesophases show a red-shift (by 7–22 nm) in emission peak maxima compared to their alcohol dispersions. This bathochromic shift may arise from the rearrangement of the hydrogen bond network between PG CNDs and the dispersing media (low-carbon alcohol or DLPC matrix). As reported by Mucha et al.,^49^ the formation of the hydrogen bonds between hydroxyl moieties of PG CNDs (hydrogen-bond donors) and the acceptor counterparts (here phosphate groups from the headgroup of PC) facilitates the transfer of electrons (from free electron pair) to CNDs. This results in new low-energy intragap states and the red-shifted emission, as compared to the alcohol dispersion case, which is a hydrogen bond donor in the suspension of PG CNDs.

Moreover, the fluorescence spectrum of the CYAN CNDs in the stack of lipid bilayers differs from that of the others. It contains two additional peaks, which may correspond to different fractions of CNDs (Figure 5b). Presumably, this is due to the high concentration of NPs in the sample. A similar situation was observed in the TPE emission spectra of CYAN CNDs (*C* = 0.5 mg/mL), while the diluted suspension of the same PG CNDs (*C* = 83.3 μg/mL) exhibits a narrow fluorescence spectrum (with FWHM ∼40 nm) (Figure S6). The aggregation-induced appearance of new emission-spectrum components has already been noted for PG CNDs.^58^ The significant increase in the concentration of PG CNDs may modify the hydrogen bond network, involve interactions between hydrogen-bond donors and acceptors of PG CNDs, and induce the aggregation of these NPs.

Then, we monitored the 3D morphology of various forms of DLPC-based MFs. The first stack of emission intensity maps shows an oval structure doped with CYAN CNDs bent in one direction (Figure 5c). We observed no lipid-free volume in the structure, which is consistent with previous observations for MFs with an oval-ended form.^53^ The following experiments focused on straight elongated structures. The hollow cross section was observed in samples doped with GREEN CNDs (Figure 5d), similar to the previously mentioned CYAN CNDs-doped MFs (Figure 4). In the sample containing YELLOW CNDs-doped MFs, we analyzed a tube with a solid cross section, *i.e*., without an explicit aqueous core, which is typical for samples originating directly from a pinned lipid droplet (Figure 5e). Moreover, regions of noticeably higher or lower intensity were not identified within the MF, indicating a lack of a preferential distribution of PG CNDs in the specific areas of the sample.

Here, we report the novel concept of combining PG CNDs with phospholipids to detect hydrophobic regions of MFs using one- and two-photon excited fluorescence microscopy. Our results indicated that mesophases containing PCs and the appropriate concentration of PG CNDs formed at a neutral pH retain the liquid crystalline properties of pristine MFs. With PLM, we demonstrated that the presence of NPs does not disturb the formation of the distinctive morphologies of the MFs. Further microscopic and spectroscopic studies proved that multicolored PG CNDs in a lipidic matrix can be excited in linear and nonlinear regimes. The presented findings showed that PG CNDs can serve as fluorescent probes to efficiently image various shapes of PC-based multilamellar microstructures. Using noninvasive and high-resolution techniques, such as TPEFM, to detect PG CNDs-doped lipidic mesophases in 3D may have significant implications in bioimaging, as even subtle changes in the lipid-rich myelin sheath can impact axonal conduction. Moreover, selecting PG CNDs with narrow emission spectra at specified spectral ranges to stain lipidic mesophases can be crucial to optimizing spectral overlap in the simultaneous imaging of multiple fluorophores. Considering that carbon-based NPs are in line with the trend of environmentally friendly materials and the excellent performance of PG CNDs, we demonstrated an appealing alternative to commercial lipophilic fluorescent molecules for exploring biologically relevant PC-based structures.

## Supporting Information Available

Experimental details (including materials, synthesis of PG CNDs, preparation of MFs, and description of setups), as well as additional spectroscopic and microscopic data (PDF)
Transparent Peer Review report available (PDF)
