# Supplementary material for: Three-Dimensional Imaging of Bioinspired Lipidic Mesophases Using Multicolored Light-Emitting Carbon Nanodots
Source: J Phys Chem Lett. 2024 Jun 11;15(24):6383–91. doi: 10.1021/acs.jpclett.4c00788 (PMC11194803; doi:10.1021/acs.jpclett.4c00788)
Supplement: Supplementary file 1 — jz4c00788_si_001.pdf [file jz4c00788_si_001.pdf]

Supporting Information

Three-Dimensional Imaging of Bioinspired Lipidic Mesophases Using Multicolored Light-emitting Carbon Nanodots

Dominika Benkowska-Biernacka[1], Sebastian G. Mucha[2], Katarzyna Matczyszyn[1, 3*]*

[1]Institute of Advanced Materials, Faculty of Chemistry, Wroclaw University of Science and Technology, ul. Wybrzeze Wyspianskiego 27, 50-370 Wroclaw, Poland

[2]Laboratoire Charles Coulomb (L2C), UMR5221, Université de Montpellier (CNRS), Campus Triolet, Place Eugene Bataillon, Montpellier 34095, France

[3]International Institute for Sustainability with Knotted Chiral Meta Matter (WPI-SKCM[2]), Hiroshima University, Higashihiroshima, Hiroshima 739-8526, Japan

*dominika.benkowska@pwr.edu.pl, katarzyna.matczyszyn@pwr.edu.pl

1. Experimental section

Materials

Synthetic phospholipids, 1,2-dilauroyl-sn-glycero-3-phosphocholine (DLPC) and 1,2 dimyristoyl-sn-glycero-3-phosphocholine (DMPC), were purchased from Avanti Polar Lipids. Phloroglucinol, boric acid, and silica gel (70–230 mesh, 63–200 mm, and pore size 60 Å) were supplied by Sigma Aldrich. High-grade purity dichloromethane, methanol (MeOH), and ethanol (EtOH) were purchased from POCH S.A. The pH of the aqueous phase was adjusted by adding the appropriate amount of HCl to Milli-Q water provided by an ultrapure water system. All chemicals were stored under proper conditions and used without further purification processes.

Synthesis of phloroglucinol-derived carbon nanodots

Phloroglucinol-derived carbon nanodots (PG CNDs) were prepared as Mucha *et al.* described,[1] resulting in three types of nanostructures with different emission colors (CYAN, GREEN, and YELLOW CNDs). Briefly, CYAN and GREEN fractions of CNDs were synthesized following the solvothermal treatment of the PG molecules in EtOH (for 3 h and 9 h at 200 ^{o}C, respectively). YELLOW CNDs were fabricated using the thermal decomposition route in molten boric acid (for 3 h at 200 ^{o}C). Each reaction mixture was then purified with the multistep procedure, involving filtration and the silica column chromatography (with dichloromethane: MeOH eluent mixture; the volume ratio of 6:1). High purity of PG CNDs was evidenced with the thin-layer chromatography and the UV-Vis absorption/conventional fluorescence spectroscopies.

Preparation of myelin figures

Phospholipid-based myelin figures (MFs) were prepared following the contact method.[2] Certain amounts of phosphatidylcholines (PCs) were dissolved in pure EtOH or ethanolic dispersions of PG CNDs at concentrations ranging from 0.1 to 1.0 mg/mL. The final concentration of the lipid

stock solution was 30 mg/mL. The stock solution was deposited by drop casting on a clean microscope slide and kept overnight under the vacuum. Then, a coverslip was placed over the dried droplet of stock solution with PG CNDs, and the liquid crystalline cell was filled with Milli-Q water. After 10 min of sample incubation at room temperature, its edges were sealed with epoxy glue. Samples prepared at acidic pH were formed after the hydration of the dried lipid droplet with an aqueous HCl solution at pH = 1.

Optical and structural characterization of PG CNDs

All one-photon excited (OPE) fluorescence spectra of PG CNDs in the dispersion form were measured on a FluoroMax-4 spectrofluorometer (Horiba Jobin Yvon) with excitation/emission slits of 3.0 nm. TPE emission spectra were recorded on a Shamrock 303i spectrometer (Andor) connected with a sensitive iDus camera (Andor). To irradiate the samples of PG CNDs, a mode-locked femtosecond Ti: Sapphire (Chameleon, Coherent Inc.) was used. The laser system operates with a pulse duration of 100 fs and a repetition rate of 80 MHz in the wavelength range of 690-1080 nm. The absolute fluorescence quantum yield of CYAN CNDs[1] in 10 mM Tris Buffer (pH = 7.4) was determined using a home-built experimental setup, consisting of an integrating sphere, a neutral density filter (Thorlabs), a high-sensitivity spectrometer (QE Pro, Ocean Optics), and a BDL - 375 - SMN Picosecond Laser Diode (377 nm) as an excitation source (Figure S7). Each spectroscopic measurement was performed at room temperature.

Transmission electron microscopy imaging was performed using a Talos F200i (Thermo Fisher Scientific) operated at 200 kV. Samples of PG CNDs were dispersed in pure EtOH to reach a concentration of 5.0 μg/mL and drop casted on a carbon-coated copper grid (Agar Scientific).

Characterization of the formation and properties of MFs doped with PG CNDs

Polarized light microscopy photographs were obtained by an Olympus BX60 optical microscope with crossed polarizers and a 530 nm retardation plate. A light source was changed from a halogen lamp to a mercury fluorescence illuminator for fluorescence imaging. Images were taken with 10x/0.30 NA and 20x/0.50 NA objectives.

3D imaging of MFs doped with PG CNDs using one- and two-photon excited fluorescence microscopy

The confocal microscopy experiments were carried out using a Leica TCS SP8 with 405 nm and 488 nm diode lasers as excitation sources. The fluorescent images were performed using 40x/0.85 NA dry and 63x/1.4 NA oil objectives. Leica Las X Software was used to acquire scans taken along the z-axis.

The mode-locked Ti: Sapphire Chameleon laser (Coherent Inc.) was used to generate femtosecond light pulses with durations around 100 fs and a repetition rate of 80 MHz. The appropriate long-pass filter was added to remove unwanted radiation; depending on excitation wavelength appropriate longpass filters (FELH700, FELH800 or FELH900 from Thorlabs) were inserted into the setup. The samples were mounted on an epi-illumination microscope inverted microscope (Nikon Eclipse Ti-U) equipped with the XYZ-positioning piezoelectric stage (Piezosystem Jena). The specimens were observed using a 40×/0.75 NA dry objective and 100x/1.4 NA oil immersion objective (Nikon Planar Fluor). The average incident laser power on the sample was adjusted using a half-wave plate and reflective neutral density filter. The fluorescence signals from the sample passed through the short-pass filter 720 nm (FF01-720/SP25) and a dichroic beam splitter (FF670-SDi01). The luminescence signals were detected by avalanche photodiodes (IDQ id100).

Statistical Analysis

The Spearman's rank correlation test was performed to evaluate the strength of nonlinear monotonic associations. The strength of correlation increases as absolute values of r_s in the range of 0 – 1. The sign of the r_s indicates the direction of the relationship between two variables.[3] The statistical significance of the correlation was determined by p-value (significance level of 0.05).[4] The dimensions of MFs used for calculations were measured from the polarized light optical images using ImageJ.

2. Supplementary figures and tables

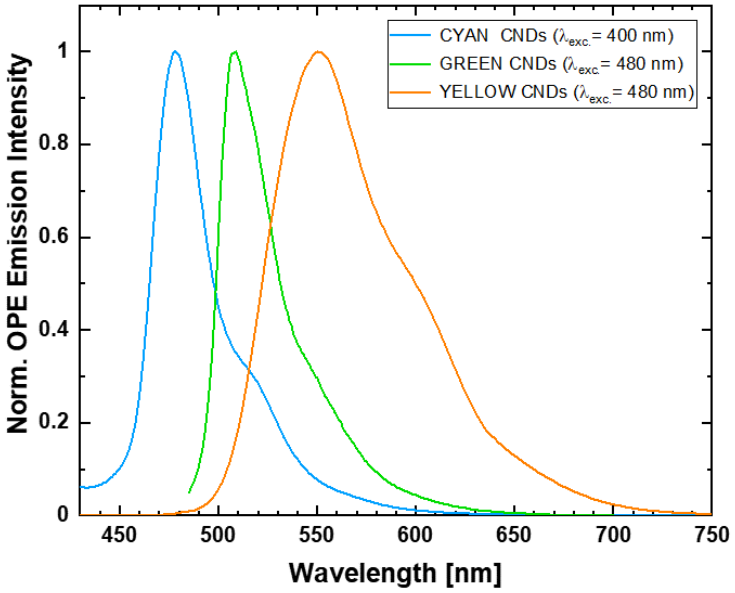

Figure S1 Normalized one-photon excited (OPE) emission spectra of multicolor emitting PG CNDs dispersed in MeOH. The samples were excited at 400 nm (CYAN CNDs) and 480 nm (GREEN and YELLOW CNDs). Materials were reproduced from Mucha *et al*. with permission from the Royal Society of Chemistry.[1]

Table S1 The fundamental optical parameters of PG CNDs in MeOH dispersions: fluorescence quantum yields (FQYs) and two-photon absorption merit factors (σ_{TPA}/M).[1]

Sample	FQY [%]	σ_{TPA}/M [GM·mol/g]
CYAN CNDs	61.8	$1.2 \cdot 10^{-3}$ (at 740 nm)
GREEN CNDs	29.7	$2.1 \cdot 10^{-3}$ (at 760 nm)
YELLOW CNDs	25.2	0.31 (at 740 nm)

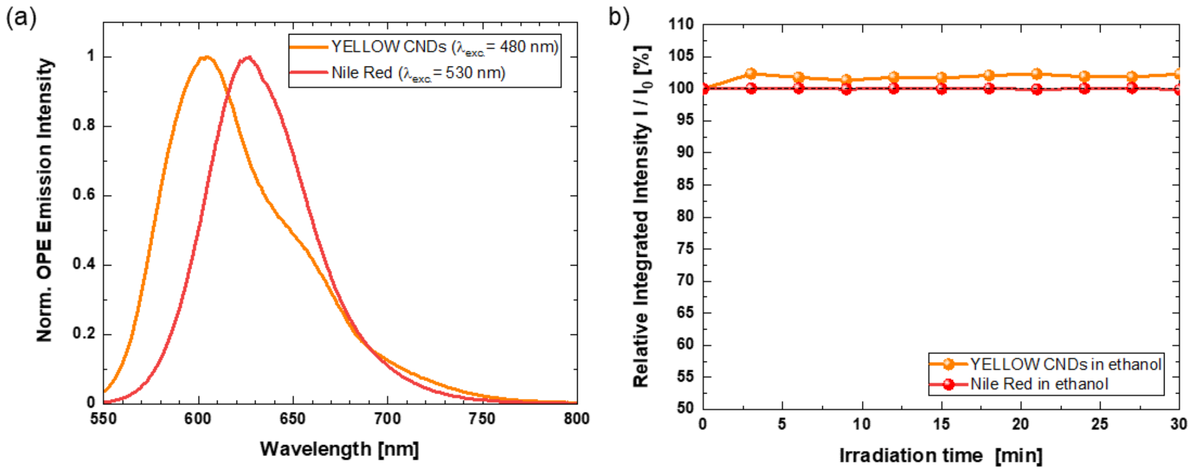

Figure S2 (a) OPE emission spectra of YELLOW CNDs (marked in orange) and Nile Red (marked in red) in EtOH. The samples were excited at 480 nm (YELLOW CNDs) and 530 nm (Nile Red). **(b)** Photostability of YELLOW CNDs and Nile Red in EtOH under continuous irradiation with the pulsed laser beam (λ = 740 nm, P_{laser} = 10 mW). YELLOW CNDs and Nile Red were excited at 480 and 530 nm, respectively. The black dashed line indicates 100% level.

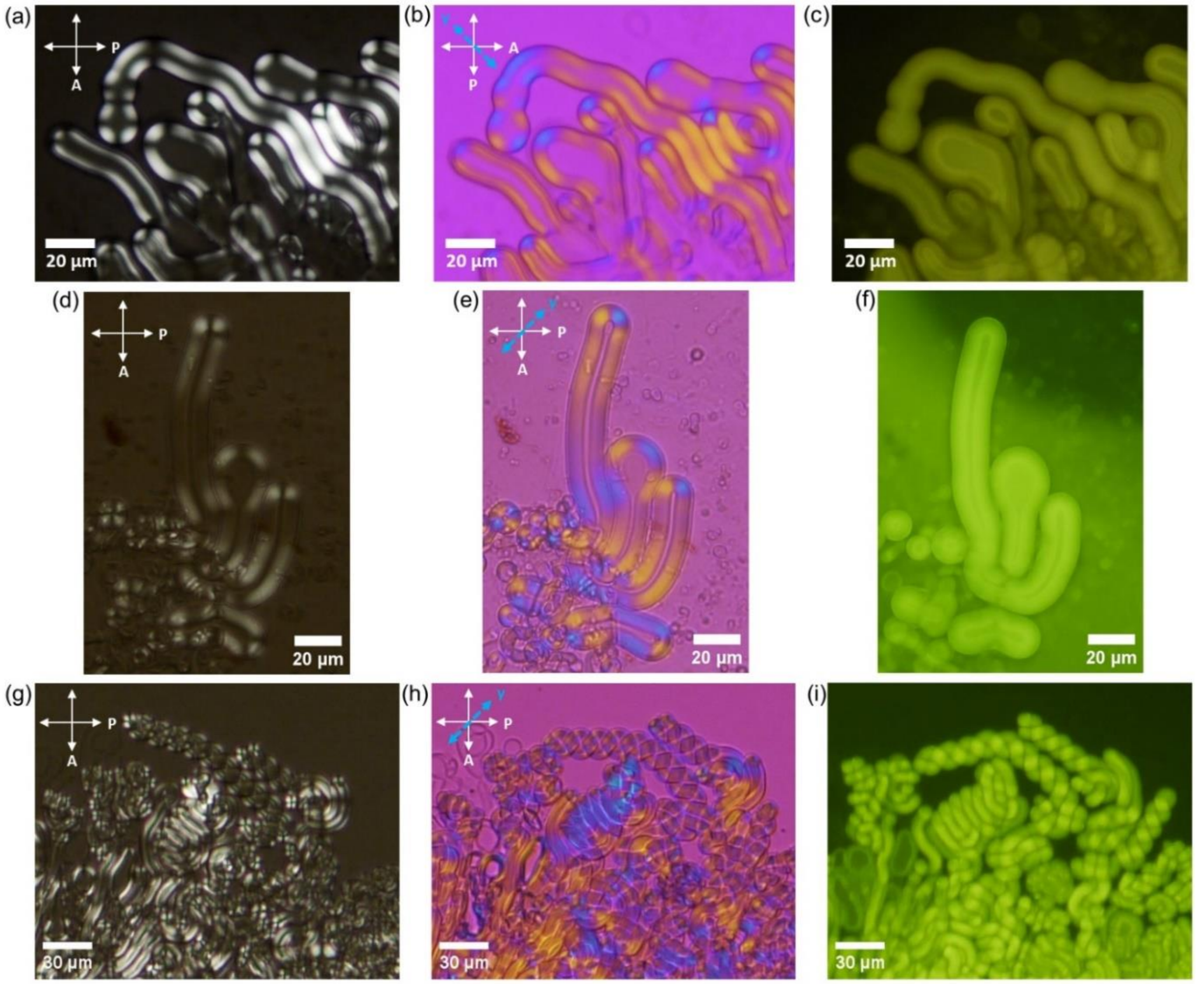

Figure S3 Imaging of different forms of MFs doped with **(a-c)** GREEN and **(d-i)** YELLOW CNDs using polarized light (left and center columns) and widefield fluorescence microscopy under excitation in the range of 460 – 495 nm (right column). The orientations of two polarizers ('A' and 'P') are indicated by white double-sided arrows and the direction of the slow axis of the retardation plate (γ) is marked as a blue double arrow. Scale bars are **(a-f)** 20 µm and **(g-i)** 30 µm.

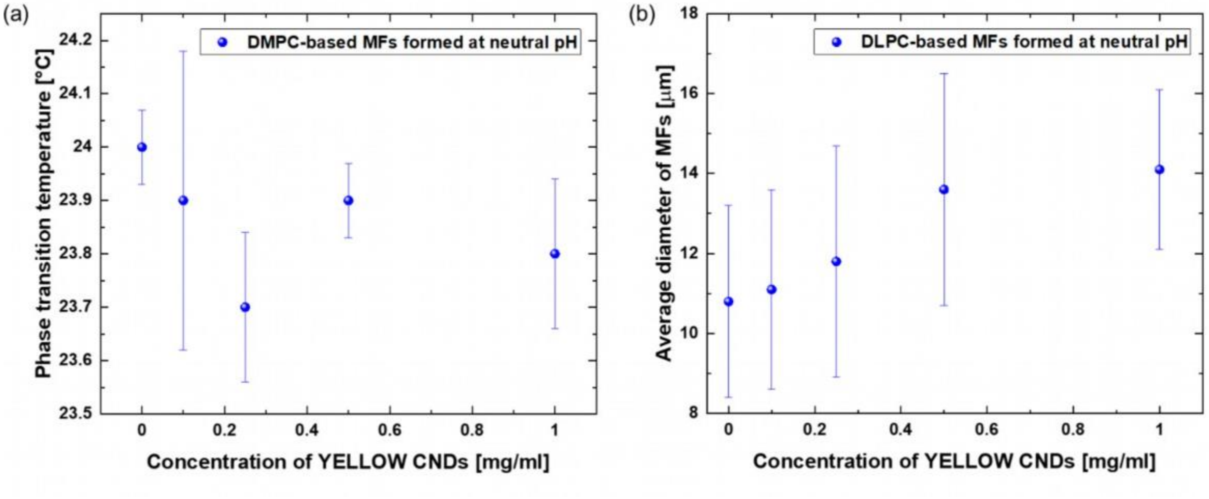

Figure S4 (a) Scatter plot indicating the formation temperature of DMPC-based MFs with and without CNDs after hydration of a dried lipid droplet with a solution at pH = 1. **(b)** Graph depicting the average diameter of DLPC-based MFs formed with and without YELLOW CNDs at acidic (red dots) and neutral (blue dots) pH.

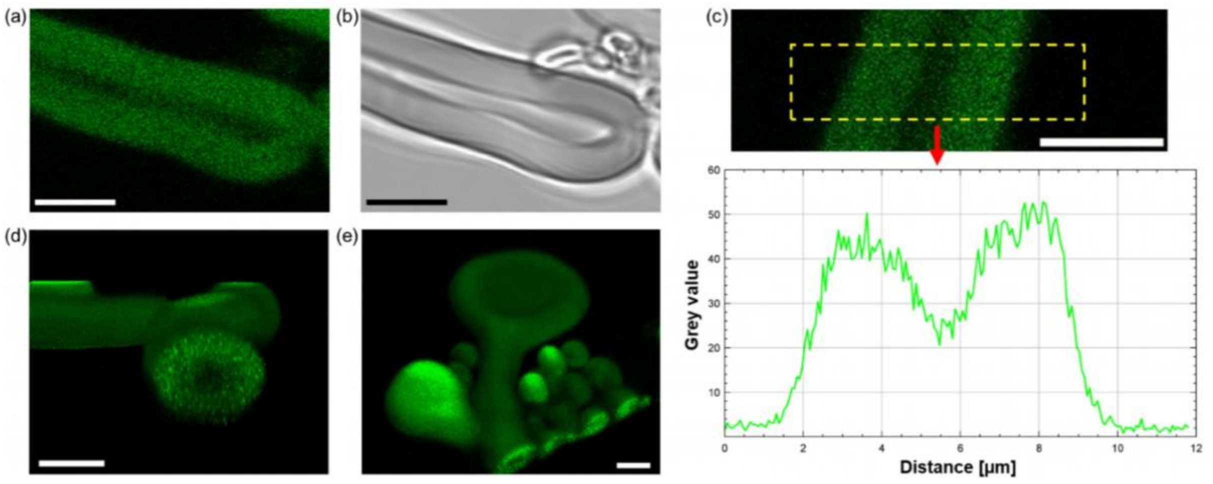

Figure S5 (a) The confocal fluorescence microscopy image of DLPC-based MFs marked with GREEN CNDs taken upon excitation at 480 nm (λ_{em}: 510 - 520 nm) and **(b)** a bright field image of the same specimen region. **(c)** The fluorescence intensity plot profile was obtained for the area (enlarged image from **(a)**) marked by a yellow square). The x-axis shows the horizontal distance within the selected area, and grey values represent vertically averaged pixel intensity. **(d, e)** The corresponding 3D morphologies of the samples are presented in **(a)** and **Figure 3b**, respectively. Scale bars are 5 µm.

Table S2 The maximum emission wavelengths of CYAN, GREEN, and YELLOW CNDs in MeOH and DLPC-based MFs under different excitation wavelengths ($\lambda_{exc.}$) from the NIR region.

Sample	Emission maximum in alcohol dispersion [nm]	Emission maximum in DLPC matrix [nm]
CYAN CNDs	480 ($\lambda_{exc.}$ = 860 nm)[1]	487 ($\lambda_{exc.}$ = 850 nm)
GREEN CNDs	514 ($\lambda_{exc.}$ = 940 nm)[1]	536 ($\lambda_{exc.}$ = 940 nm)
YELLOW CNDs	551 ($\lambda_{exc.}$ = 740 nm)[1]	566 ($\lambda_{exc.}$ = 740 nm)

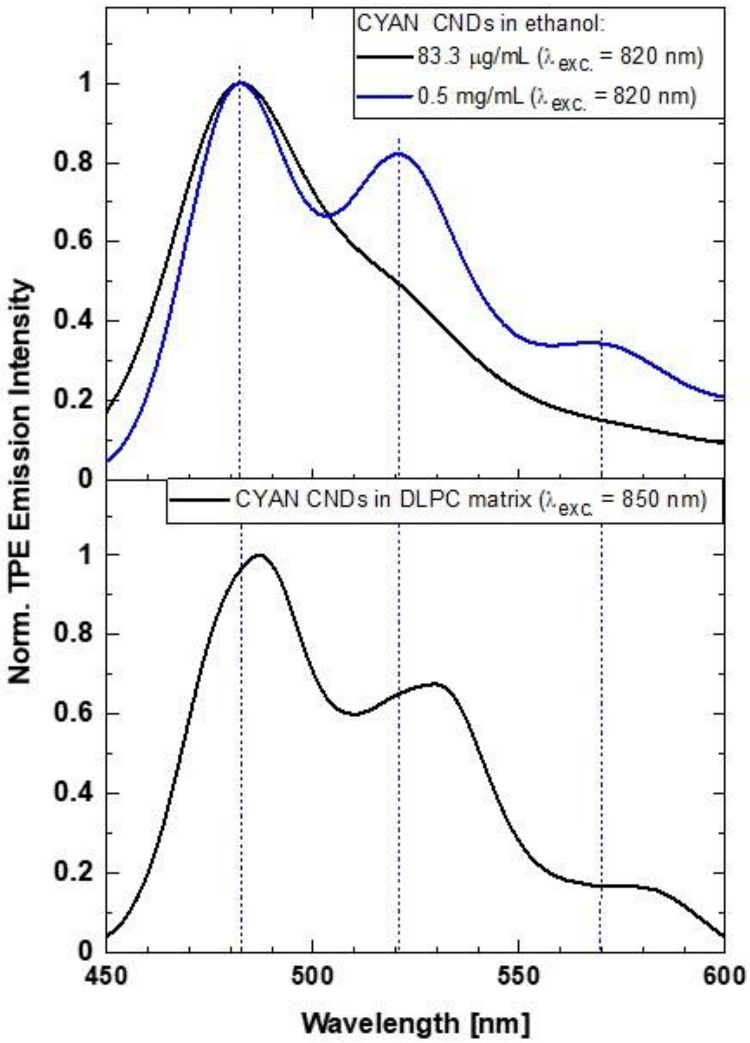

Figure S6 (top) Two-photon excited (TPE) emission spectra of CYAN CNDs dispersed in ethanol at 83.3 mg/mL and 0.5 mg/mL ($\lambda_{exc.}$ = 820 nm). The dashed lines indicate the emission maxima of CNDs at higher concentrations. **(bottom)** TPE emission spectra of CYAN CNDs in DLPC matrix ($\lambda_{exc.}$ = 850 nm).

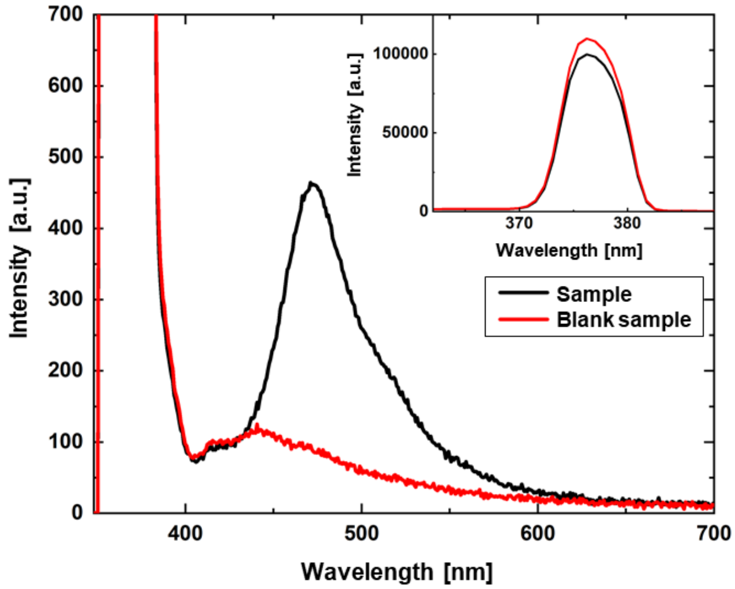

Figure S7 The one-photon excited fluorescence spectra of CYAN CNDs in 10 mM Tris Buffer (pH = 7.4; sample) and the corresponding blank sample ($\lambda_{exc.}$ = 377 nm).

REFERENCES

(1) Mucha, S. G.; Firlej, L.; Formalik, F.; Bantignies, J.-L.; Anglaret, E.; Samoc, M.; Matczyszyn, K., Revealing two chemical strategies to tune bright one- and two-photon excited fluorescence of carbon nanodots. *J. Mater. Chem. C* **2024,** *12* (6), 2117-2133, DOI: 10.1039/D3TC03211F.

(2) Zou, L.-N., Myelin figures: The buckling and flow of wet soap. *Phys. Rev. E* **2009,** *79* (6), 061502, DOI: 10.1103/PhysRevE.79.061502.

(3) Akoglu, H., User's guide to correlation coefficients. *Turk. J. Emerg. Med.* **2018,** *18* (3), 91-93, DOI: 10.1016/j.tjem.2018.08.001.

(4) Di Leo, G.; Sardanelli, F., Statistical significance: p value, 0.05 threshold, and applications to radiomics-reasons for a conservative approach. *Eur. Radiol. Exp.* **2020,** *4* (1), 18, DOI: 10.1186/s41747-020-0145-y.
