# Supplementary material for: Three-Dimensional Imaging of Bioinspired Lipidic Mesophases Using Multicolored Light-Emitting Carbon Nanodots
Source: J Phys Chem Lett. 2024 Jun 11;15(24):6383–91. doi: 10.1021/acs.jpclett.4c00788 (PMC11194803; doi:10.1021/acs.jpclett.4c00788)
Supplement: Supplementary file 2 — jz4c00788_si_002.pdf [file jz4c00788_si_002.pdf]

jz-2024-00788e.R1

Name: Peer Review Information for "Three-Dimensional Imaging of Bioinspired Lipidic Mesophases Using Multicolored Light-Emitting Carbon Nanodots."

First Round of Reviewer Comments

Reviewer: 1

Comments to the Author

In this manuscript, the authors demonstrated the possibility of selective labeling of MFs by biocompatible polychromic phloroglucinol-derived carbon nanodots (PG-CNDs). The three-dimensional structure of MFs was obtained by combining single photon and two-photon fluorescence microscopy. This design idea is interesting and the test results are detailed. I recommend that it should be accepted to be published after the following issues have been addressed.

1) Nile red is a lipophilic fluorescent dye and therefore has a strong affinity for fats and lipids. The authors believe that PG-CNDs is also a lipophilic fluorescent molecule. However, PG-CNDs is rich in hydroxyl structure, which will increase the hydrophilicity of PG-CNDs. How do the authors prove the lipophilicity of PG-CNDs?

2) In this manuscript, the photostability of PG-CNDs is crucial for imaging MFs, which should be described and compared in detail.

3) Although CDs are thought to have lower cytotoxicity, making them more promising for long-term imaging and live cell imaging. However, the authors still need to conduct toxicity related experiments for PG-CNDs, which are not covered in this manuscript and the author's recent article (J. Mater. Chem. C, 2024,12, 2117-2133).

4) In conclusion, the authors suggest that PG-CNDs is an alternative to commercial lipophilic fluorescent molecules. However, in the manuscript, this conclusion cannot be drawn due to the lack of performance comparison with conventional fluorescent lipid materials. What are the outstanding advantages of PG-CNDs over Nile red and other traditional fluorescent lipid dyes?

Reviewer: 2

Comments to the Author

This article “Three-Dimensional Imaging of Bioinspired Lipidic Mesophases Using Multicolored Light-emitting”, the authors demonstrated the possibility of selective labeling of MFs by biocompatible and strongly emitting multicolor phloroglucinol-derived carbon nanodots (PG CNDs), and proved that PG CNDs can be a viable and simple alternative to conventional fluorescent lipid stains to image biologically relevant phospholipid-based structures. I would like to recommend to publish this article on “J. Phys. Chem. Lett.”. But please be noted there are some details need to be further explained and revised.

1. The author reported the concept of combining PG CNDs with phospholipids to detect hydrophobic regions of MFs using one- and two-photon excited fluorescence microscopy. However, the manuscript mainly discussed the formation of MFs and the imaging capabilities of CNDs. Almost nothing besides the optical properties were mentioned about the properties and characterization of CNDs. As the key material described in the article, the properties of CNDs are almost completely unknown, the author needs to supplement the following basic characterizations about CNDs.

2. Characterize the particle size and height of CNDs by TEM and AFM. Can the lattice fringes in the carbon core be observed through TEM?

3. Characterize the chemical structure and elemental composition by FT-IR and XPS, and what is the origin of the photoluminescence of CNDs?

4. How about the biological toxicity of these CNDs? Authors need to provide MTT cytotoxicity experimental data.

5. What is the difference between CNDs and commercial dyes? More explanation on the advantage of CND should be given.

6. As the imaging application of CNDs is in water media. The PLQYs of these CNDs in water should be given.

Reviewer: 3

Comments to the Author

In this manuscript, Matczyszyn and coworkers reported labelling of MFs by biocompatible and strongly emitting multicolour phloroglucinol-derived carbon nanodots (PG CNDs) using fluorescence microscopy and spectroscopy techniques along with polarized light microscopy. The authors reported the stability and morphology of lipidic mesophases. The author claimed that PG

CNDs can be a viable and simple alternative to conventional fluorescent lipid stains to image biologically relevant phospholipid-based structures.

The manuscript may be publishable in JPC Letters, However, I am not convinced by the urgency of the publication. So, I am recommending a major revision for this manuscript. The authors should address the following issues before publications.

1. A similar work has been recently published by the authors (J. Phys. Chem. B 2020, 124, 11974−11979). Thus, the claims in the manuscript are not very new. The auhors should explain in detail the additional advantage of taking carbon dots? The manuscript lacks new insight regarding the interaction between myelin and lipid bilayer.

2. It is not clear why the authors chose PG CNDs. There are a large numbers of carbon dots available in the literature which are often used for fluorescence and imaging. The PG CNDs are not exceptional. I expect that the other carbon dots with wide range of emission will exhibit a similar morphology like the PG CNDs do. The author needs to do the experiment with at least one conventional CNDs to substantiate the superiority of PG CNDs

3. The author mainly worked with the zwitterionic lipid. What would be the effect of ionic lipid on the MF morphology? All the experiments were conducted in Mili-Q water, is there is any role of buffer in governing the interaction between lipid and Myelin.

Although the authors emphasized on the morphology of MF, it would be interesting to get the morphology using different lipid at different condition.

4. It would be better if images of blank liposome by PG CND is shown. The exact location of PG CNDs is not well understood in the manuscript.

5. In figure 5, we observed that there is a redshift in the emission of CNDs in presence of lipid. The author mentioned that hydrogen bonding is responsible for the observed shift. I was curious whether this kind of CND undergo aggregation in presence of lipid molecules. The emission may come from the aggregated form. The author needs to address this issue.

6. The author claimed that the diameters of the DMPC-based MFs were enhanced at acidic and neutral pH. They attributed to this protonation of phosphate group. What would be impact on the diameter of the tube if a lipid of lower headgroup is used (with higher phase transition temperature)?

7. The authors need to confirm whether CDN donot perturb the morphology of the lipid bilayer. The location of CDN is very important. It needs to be confirmed if CDN undergo homogeneous distribution in the lipid bilayer surface.

8. A new insight may be given regarding the interaction of myelin and lipid bilayer interaction.

Author's Response to Peer Review Comments:

Wroclaw, 28.04.2024

Prof. Katarzyna Matczyszyn
Wroclaw University of Science and Technology,
Wyb. Wyspianskiego 27
50-370 Wroclaw, Poland
katarzyna.matczyszyn@pwr.edu.pl

Dear Editor

On behalf of all co-authors, thank you for the consideration of publishing our manuscript:

Three-Dimensional Imaging of Bioinspired Lipidic Mesophases Using Multicolored Light-emitting Carbon Nanodots

by Dominika Benkowska-Biernacka, Sebastian G. Mucha, and Katarzyna Matczyszyn.

After careful evaluation by the reviewers, our manuscript received major and minor revisions. As suggested, we have revised the manuscript, following all comments provided by reviewers. A detailed list of modifications, remarks, and responses to the reviewers' feedback is given below. Note that all text modifications in the main body and the Supporting Information are marked with blue font.

We hope that the revised manuscript and our replies will meet your requirements, enhance the quality of our manuscript, and enable publication.

Kind regards,

Katarzyna Matczyszyn

Editors' Remarks

1. Please include annotated version(s) of your revised publication file(s) with colored text or highlights indicating the revisions that you have made, and upload them as "Supporting Information for Review Only." Please also upload "clean" copies for publication. (No highlighting, annotations, or colored text permitted.)

2. Title: In both the main manuscript file and the Supporting Information, set the title in title case, with the first letter of each principal word capitalized.

3. Title and Author Lists: Title, author names, and affiliations must match in three places: (1) manuscript file, (2) supporting information, and (3) ACS Paragon Plus. See the Supportining Information affiliations and labels.

4. References: In both the main file and the supporting information, fix the style of all references to use JPCL formatting (check all references carefully). ***JPC Letters reference formatting requires that journal references should contain: () around numbers; author names; article title (titles entirely in title case or entirely in lower case); abbreviated journal title (italicized); year (bolded); volume (italicized); and pages (first-last). Book references should contain author names; book title (in the same pattern); publisher; city; and year. Websites must include date of access.

Authors' reply: Thank you for your comments. We have made the necessary revisions to the manuscript and Supporting Information.

Reviewer: 1

Recommendation:

This paper is publishable subject to minor revisions noted. Further review is not needed.

Comments:

In this manuscript, the authors demonstrated the possibility of selective labeling of MFs by biocompatible polychromic phloroglucinol-derived carbon nanodots (PG-CNDs). The three-dimensional structure of MFs was obtained by combining single photon and two-photon fluorescence microscopy. This design idea is interesting and the test results are detailed. I recommend that it should be accepted to be published after the following issues have been addressed.

1. Nile red is a lipophilic fluorescent dye and therefore has a strong affinity for fats and lipids. The authors believe that PG-CNDs is also a lipophilic fluorescent molecule. However, PG-CNDs is rich in hydroxyl structure, which will increase the hydrophilicity of PG-CNDs. How do the authors prove the lipophilicity of PG-CNDs?

Authors' reply: Thank you for bringing this matter to our attention. Based on numerical and experimental studies presented in the previous article on PG CNDs, apart from oxygenous groups (*e.g.* hydroxyls), these nanoparticles are composed of a variety of sp^2- and sp^3-hybridized carbon groups.[1] More precisely, the spectral characteristics of PG CNDs (*i.e.* provided by infrared (IR) and nuclear resonance magnetic spectroscopies) show a large number of intense peaks that can be assigned to various non-polar methyl and methylene moieties with their essential role. Moreover, the quantitative analysis (from the X-ray photoelectron spectroscopy, XPS) indicates the predominant contribution of these non-polar moieties (as compared to polar ones) in CYAN and GREEN and a comparable contribution in YELLOW CNDs. More details on the structure of PG CNDs are described in our previous article.[1] Phosphatidylcholines, used in our paper as the main component of MFs, consist of a polar phosphate head group and two non-polar aliphatic chains. As a result, interactions between non-polar components of both PG CNDs and MFs are likely expected. The manuscript has been supplemented.

Furthermore, we have confirmed that CNDs label the hydrophobic parts of the myelin figures and do not accumulate in their water cores. These results can be compared with results obtained for phospholipid-based MFs marked with hydrophilic folic acid-based CNDs, which stained the inner channel running along the MFs and water layers alternating phospholipid bilayers **(Figure 5 and R1)**. [2]

Page 6, line 15:

Furthermore, PG CNDs consist of polar (e.g. hydroxyl groups) and non-polar (methyl and methylene moieties) structural units. Their extensive structural characterization unravelled the predominant role of non-polar carbogenic domains. (49)

Page 3, line 23:

Moreover, carbon nanodots (CNDs) have been successfully applied in staining the aqueous phase in multilamellar lipid tubes.(30) However, there is still a lack of studies on doping hydrophobic regions of MFs with NPs and imaging lipidic mesophases with fluorescent probes offering multicolor emission upon excitation in biological windows.(31)

Page 11, line 4:

As shown using the example of MFs with GREEN CNDs, an elongated internal non-fluorescent volume could be observed along the lipid tube (Figure S5a-d). According to previous findings for samples stained with commercial dyes (Texas Red and Nile Red),(53, 55) the distinct region inside the MFs is a lipid-free water core.

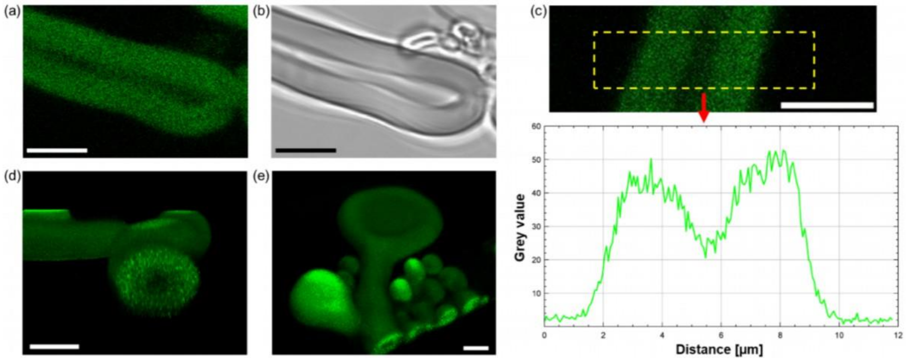

Figure S5 ***(a)*** *The confocal fluorescence microscopy image of DLPC-based MFs marked with GREEN CNDs taken upon excitation at 480 nm (λ_{em}: 510 - 520 nm) and* ***(b)*** *a bright field image of the same specimen region.* ***(c)*** *The fluorescence intensity plot profile was obtained for the area (enlarged image from* ***(a)****) marked by a yellow square). The x-axis shows the horizontal distance within the selected area, and grey values represent vertically averaged pixel intensity.* ***(d, e)*** *The corresponding 3D morphologies of the samples are presented in* ***(a)*** *and* ***Figure 3b****, respectively. Scale bars are 5 μm.*

Myelin figures with folic-acid based CNDs
(ACS Appl. Mater. Interfaces 2023, 15, 32717–32731):

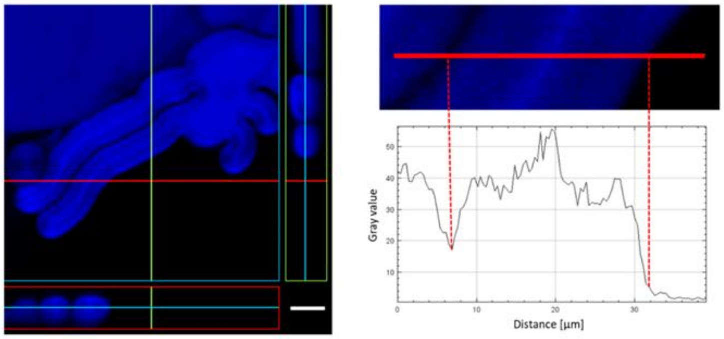

Figure R1 The confocal fluorescence microscopy images of the MFs stained with folic-acid based CNDs with cross-sectional views and **a** line profile of pixel intensity taken along the red line of the CFM image shown above. The scale bars represent 10 µm. Images are reproduced from our previous article.[2]

2. In this manuscript, the photostability of PG-CNDs is crucial for imaging MFs, which should be described and compared in detail.

Authors' reply: Thank you for pointing this out. We have presented the suggested content in the manuscript andSupporting Information (**Figure S2**, the elaboration of this aspect is in the answer to the next question)

Page 7, line 9:

*As illustrated by the example of YELLOW CNDs in comparison with Nile Red (**Figure S2b**), CNDs exposed to continuous NIR-irradiation with the pulsed laser beam (λ = 740 nm, P_{laser} = 10 mW) also maintain high fluorescence intensity.*

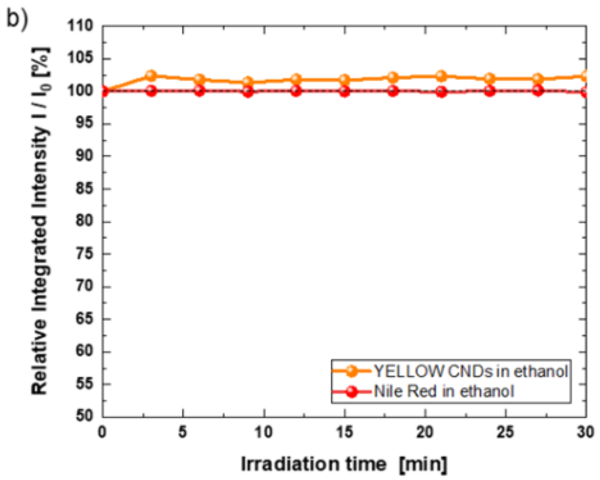

Figure S2 (b) *Photostability of YELLOW CNDs and Nile Red in EtOH under continuous irradiation with the pulsed laser beam (λ = 740 nm, P_{laser} = 10 mW). YELLOW CNDs and Nile Red were excited at 480 and 530 nm, respectively. The black dashed line indicates 100% level.*

3. Although CDs are thought to have lower cytotoxicity, making them more promising for long-term imaging and live cell imaging. However, the authors still need to conduct toxicity related experiments for PG-CNDs, which are not covered in this manuscript and the author's recent article (J. Mater. Chem. C, 2024,12, 2117-2133).

Thank you for your suggestion. We have conducted a cytotoxicity test on bone marrow-derived macrophages (BMDM) using the MTT assay. Due to the limited water solubility, nanoparticles were dispersed in MiliQ water at 5, 10, and 20 µg/mL. The cell survival rate exceeded 45 % at an initial nanoparticle concentration of 5 µg/mL (**Figure R2**). Our investigation showed that the application of PG CNDs do not disturb the formation of various shapes of MFs, which are also characteristic for pristine phosphatidylcholine-based MFs. Further research could be expanded to include more detailed cytotoxicity analyses; however, as we are not specialists in this field, another collaboration will be established to conduct them more precisely.

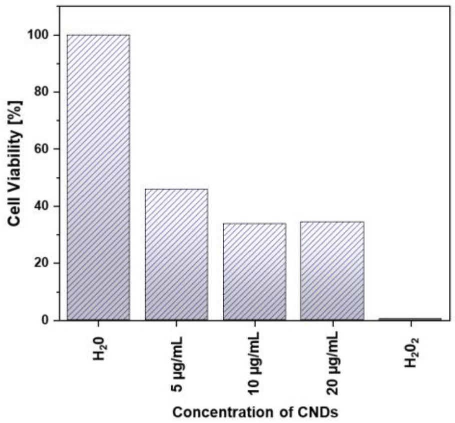

Figure R2 Cytotoxicity studies of YELLOW CNDs on BMDM cell lines.

4. In conclusion, the authors suggest that PG-CNDs is an alternative to commercial lipophilic fluorescent molecules. However, in the manuscript, this conclusion cannot be drawn due to the lack of performance comparison with conventional fluorescent lipid materials. What are the outstanding advantages of PG-CNDs over Nile red and other traditional fluorescent lipid dyes?

Authors' reply: Thank you for pointing this out. The typical stains for lipids are Nile Red and BODIPY. These common dyes have some drawbacks. For example, Nile Red shows broad emission spectra, while BODIPY exhibits limited photostability and a small Stokes shift. [3-4] We have compared the optical properties of PG CNDs with Nile Red. CYAN and GREEN CNDs show narrower emission peaks than Nile Red, with full width at half maximum (FWHM) of around 32 nm (CYAN), 33 nm (GREEN), and 62 nm (Nile Red), as presented in **Figure S1 and S2a**. Narrower FWHM results in higher spectral resolution and reduced spectral overlap with other fluorescent probes, which can be essential in bioimaging. Furthermore, both Nile Red and YELLOW CNDs show high photostability under the NIR irradiation with the pulsed laser beam (**Figure S2b**). The results mentioned above have been presented in both the main text and SI.

Moreover, the possibility of selecting an appropriate fraction of PG CNDs (with specific emission and excitation spectra, as well as, similar size), which effectively mark lipidic mesophases, allows for the development of complex imaging. For example, this approach facilitates designing and optimizing methods for simultaneous monitoring of multiple targets with several fluorescent probes in a single experiment. Despite the advantages associated with the application of multicolored nanoparticles, there is a lack of research on this topic concerning the imaging of multilamellar elongated PC-based microstructures of biological relevance. We point out this fact in the introduction.

Page 7, line 5:

Compared to the commercial lipophilic stain Nile Red (FWHM ~62 nm, Figure S2a), CYAN and GREEN CNDs exhibit narrower emission spectra. In the case of YELLOW CNDs, the FWHM value is comparable with Nile Red's value. Moreover, these yellow-emitting NPs have a significant Stokes shift (3186 cm^{-1}), which may benefit multiplex imaging.(49, 51-52) Furthermore, PG CNDs show excellent photostability under prolonged illuminations. As illustrated by the example of YELLOW CNDs in comparison with Nile Red (Figure S2b), CNDs exposed to continuous NIR-irradiation with the pulsed laser beam (λ = 740 nm, P_{laser} = 10 mW) also maintain high fluorescence intensity.

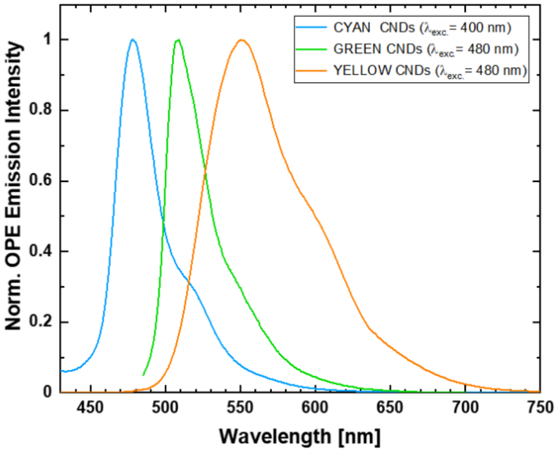

Figure S1 *Normalized one-photon excited (OPE) emission spectra of multicolor emitting PG CNDs dispersed in methanol. The samples were excited at 400 nm (CYAN CNDs) and 480 nm (GREEN and YELLOW CNDs). Materials were reproduced from Mucha et al. with permission from the Royal Society of Chemistry.*[1]

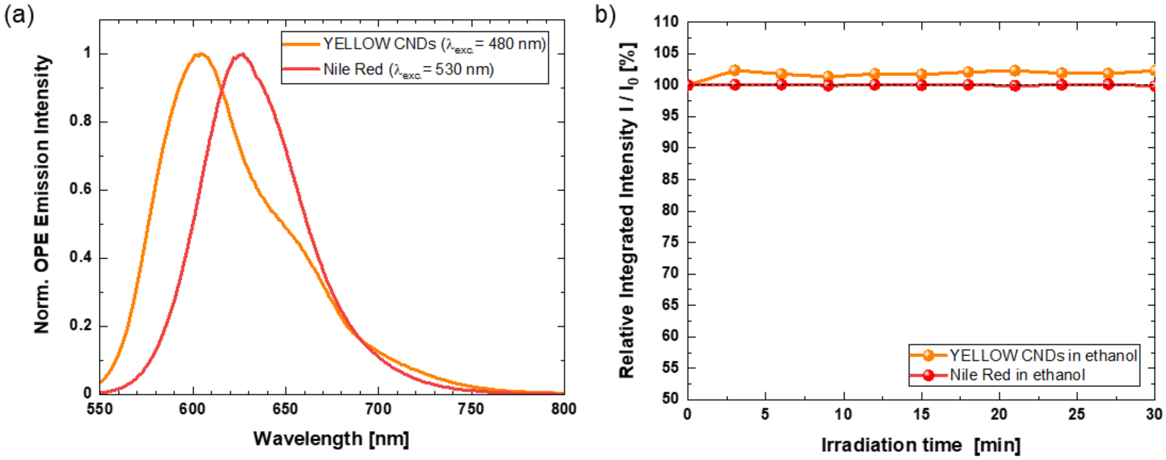

Figure S2 (a) *OPE emission spectra of YELLOW CNDs (marked in orange) and Nile Red (marked in red) dispersed in EtOH. The samples were excited at 480 nm (YELLOW CNDs) and 530 nm (Nile Red).* ***(b)*** *Photostability of YELLOW CNDs and Nile Red in EtOH under continuous irradiation with the pulsed laser beam (λ = 740 nm, P_{laser} = 10 mW). YELLOW CNDs and Nile Red were excited at 480 and 530 nm, respectively. The black dashed line indicates 100% level.*

Reviewer: 2

Recommendation:

This paper may be publishable, but major revision is needed; I would like to be invited to review any future revision.

Comments:

This article "Three-Dimensional Imaging of Bioinspired Lipidic Mesophases Using Multicolored Light-emitting", the authors demonstrated the possibility of selective labeling of MFs by biocompatible and strongly emitting multicolor phloroglucinol-derived carbon nanodots (PG CNDs), and proved that PG CNDs can be a viable and simple alternative to conventional fluorescent lipid stains to image biologically relevant phospholipid-based structures. I would like to recommend to publish this article on "J. Phys. Chem. Lett.". But please be noted there are some details need to be further explained and revised.

1. The author reported the concept of combining PG CNDs with phospholipids to detect hydrophobic regions of MFs using one- and two-photon excited fluorescence microscopy. However, the manuscript mainly discussed the formation of MFs and the imaging capabilities of CNDs. Almost nothing besides the optical properties were mentioned about the properties and characterization of CNDs. As the key material described in the article, the properties of CNDs are almost completely unknown, the author needs to supplement the following basic characterizations about CNDs.

Author reply: Thank you for the excellent suggestion. We have supplemented the manuscript with information on the morphology and structural properties of PG CNDs. We have added two high-resolution transmission electron microscopy images to **Figure 2** (the elaboration of this aspect is in answer to the next question). We also encourage the readers to learn about pure PG CNDs from our previous paper.[1] That work is devoted to the synthesis and the extensive characterization of those nanomaterials, from structural and optical points of view; it is intended to describe clearly and precisely novel nanomaterials before their use (*e.g.* in imaging of myelin figures).

Page 6, line 10:

The detailed structural and optical characterizations of applied PG CNDs (including elaboration on fluorescence mechanisms) were presented in the previous paper [1]. Based on high-resolution transmission electron microscopy analysis, PG CNDs have irregular shapes and average diameters of ~ 4 nm (exemplary results are in Figure 2a). As illustrated in Figure 2b, investigated PG CNDs show lattice spacing of ~0.32 nm (similar to the interlayer distance of 0.34 nm for the (002) plane in graphitic materials).(50) Furthermore, PG CNDs consist of polar (e.g. hydroxyl groups) and non-

polar (methyl and methylene moieties) structural units. Their extensive structural characterization unravelled the predominant role of non-polar carbogenic domains. (49)

2. Characterize the particle size and height of CNDs by TEM and AFM. Can the lattice fringes in the carbon core be observed through TEM?

Author reply: Thank you for bringing this issue to our attention. According to the size distribution of CNDs estimated based on HR-TEM images presented in a previous paper by Mucha et al., the average diameters of CYAN and GREEN CNDs were 3.9 ± 0.9 nm and YELLOW 4.3 ±0.8 nm.[1] The HR-TEM imaging was performed through the MEA Platform at Universite de Montpellier. We have performed additional HR-TEM imaging for YELLOW CNDs at Wroclaw University of Science and Technology. As shown in **Figure 2b**, HR-TEM images reveal lattice fringes in PG CNDs typical for nanomaterials with graphitic domains, as we suggested before. Based on the analysis of 50 objects of YELLOW CNDs, we confirmed their average size of ~4 nm (4.0 ± 0.7) with that result from the previous publication. The manuscript has been supplemented with TEM images and their descriptions.

Page 6, line 12:

Based on high-resolution transmission electron microscopy analysis, PG CNDs have irregular shapes and average diameters of ~ 4 nm (exemplary results are in Figure 2a). As illustrated in Figure 2b, investigated PG CNDs show lattice spacing of ~0.32 nm (similar to the interlayer distance of 0.34 nm for (002) plane in graphitic materials).(50)

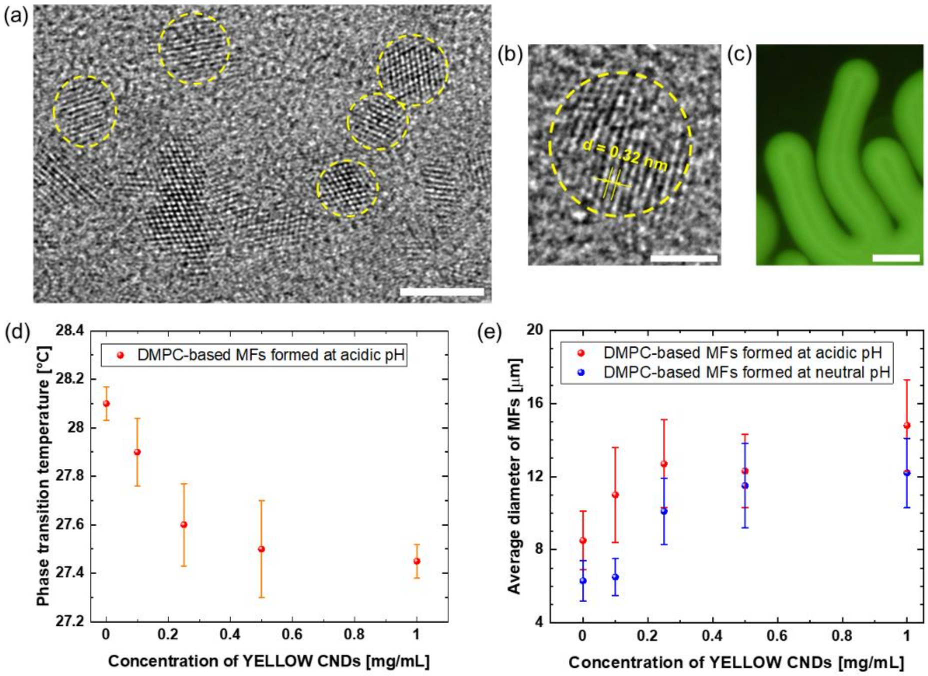

Figure 1 (a-b) *TEM images of YELLOW CNDs.* ***(c)*** *Widefield fluorescence microscopy image of MFs with YELLOW CNDs (at initial nanoparticle concentration of 1 mg/mL) formed at acidic pH ($\lambda_{exc.}$: 460 – 495 nm). Scale bars are* ***(a)*** *5 nm,* ***(b)*** *2 nm and* ***(c)*** *20 µm.* ***(d)*** *Scatter plot indicating the temperature at which DMPC-based MFs start to form with and without CNDs after hydration of a dried lipid droplet with a solution at pH = 1.* ***(e)*** *Graph depicting the average diameter of DMPC-based MFs formed with and without YELLOW CNDs at acidic (red dots) and neutral (blue dots) pH.*

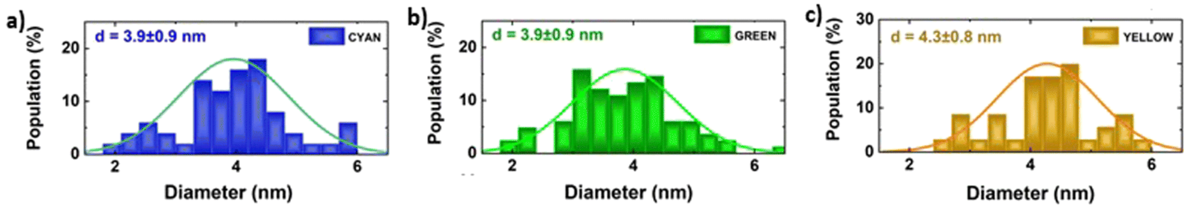

Figure R2 Size distribution of the PG CNDs. Materials were reproduced from Mucha *et al*. with permission from the Royal Society of Chemistry.[1]

3. Characterize the chemical structure and elemental composition by FT-IR and XPS, and what is the origin of the photoluminescence of CNDs?

Author reply: The origin of the photoluminescence of PG CNDs was the topic of the previous paper by Mucha et al.[1] As in other CNDs of high graphitic content, the fluorescence of PG CNDs is expected to originate from the radiative recombination of the exciton. Two chemical strategies were identified and described to affect the energy of this transition:

- the content of the oxygenous groups with the conjugation of aromatic domains,
- the donor/acceptor character of the solvating mediain the hydrogen-bond formation with PG CNDs,

Both strategies regulate the content of the π- and n-electrons within the structure of CNDs and the narrowing midgap states/formation of the intraband states. Hence, they govern the energy of the particular emission process and in consequence, the final emission color of the CNDs.

The chemical structure of PG CNDs was extensively characterized in the previous paper through different spectroscopic techniques. For example, the structural sub-units of CNDs were identified with infrared spectroscopy, operating in attenuated-total reflectance and transmission modes; both middle and far-infrared ranges were explored. The formed bonds and their contribution in particular CNDs were examined with X-ray photoelectron spectroscopy. The essential results from the main body and the Supporting Information of the previous paper are shown below.

Both the emission mechanism and description of a chemical structure were reported in the previous paper which was dedicated to them.[1]

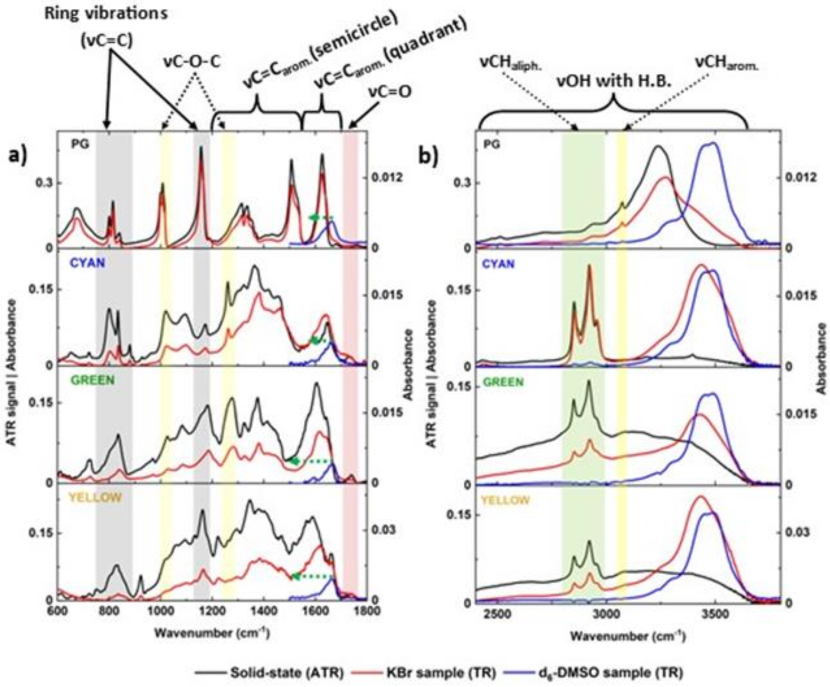

Figure R3 The FTIR spectra of PG molecules and PG CNDs in the middle-infrared range. Materials were reproduced from Mucha *et al*. [1]

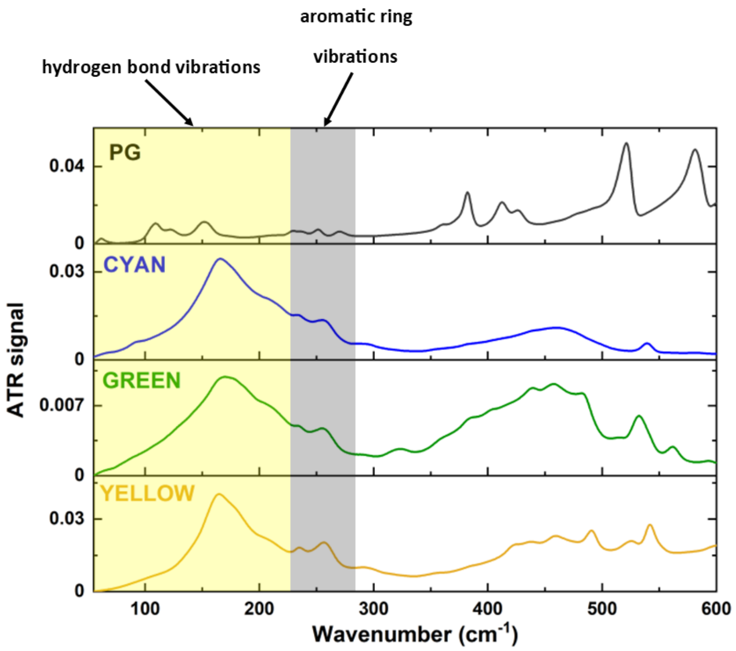

Figure R4 The FTIR spectra of PG molecules and PG CNDs in the far-infrared range. Materials were reproduced from Mucha *et al*.[1]

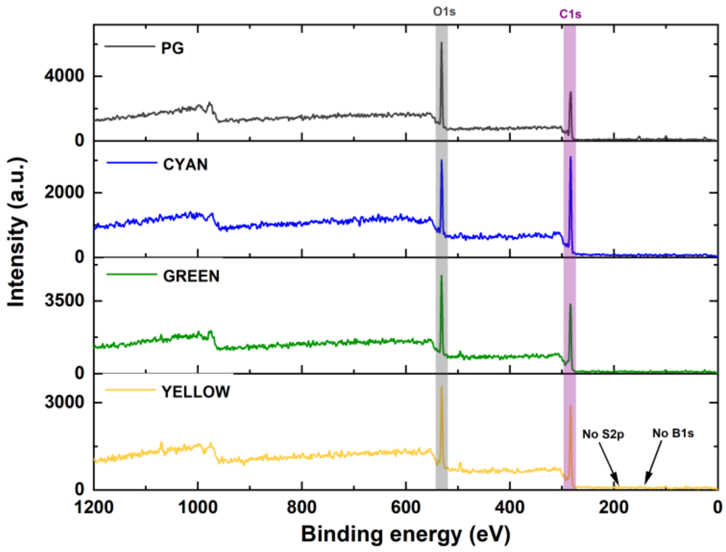

Figure R5 The XPS surveys of the PG and PG CNDs. Both crucial peaks (O1s and C1s) are indicated. Note that all CNDs are free of catalyst impurities. Materials were reproduced from Mucha *et al*.[1]

4. How about the biological toxicity of these CNDs? Authors need to provide MTT cytotoxicity experimental data.

Author reply: Thank you for your pointing it out. We have performed a cytotoxicity test on bone marrow-derived macrophages (BMDM). Experiments were carried out using the MTT assay. Since YELLOW CNDs show poor water solubility, they were dispersed in MiliQ water at concentrations of 5, 10, and 20 µg/mL. The percentage survival of cells was found to be more than 45 % at an initial concentration of nanoparticles µg/mL (**Figure R6**). PG CNDs show excellent photoluminescence performance and the tested concentration of dopants is sufficient to detect emission within mesophases. In our research, we demonstrate that the application of PG CNDs allows for the visualization of the morphology of MFs. According to the procedure of MFs preparation, the ethanolic dispersions of PG CNDs is added to the lipids before depositing them on the glass substrate. We show that the presence of CNDs in lipidic mesophases does not disturb the formation of the distinctive morphologies of MFs. Considering studies on more complex phospholipid-based models, further research should be expanded to include detailed cytotoxicity analyses (e.g., more repeats and experiments on different cell lines would be beneficial). Nevertheless, since we are not specialists in biology and we have limited experience in this field, we intend to establish a new collaboration to conduct more comprehensive studies.

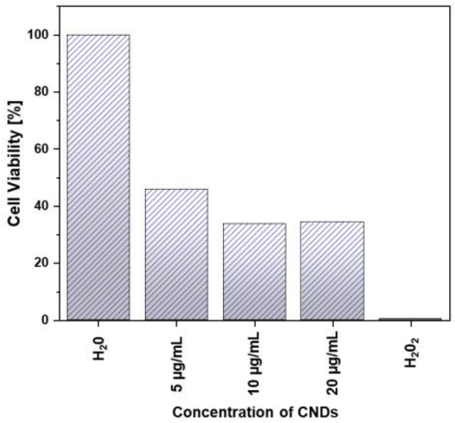

Figure R6 Cytotoxicity studies of YELLOW CNDs on BMDM cell lines.

5. What is the difference between CNDs and commercial dyes? More explanation on the advantage of CND should be given.

Author reply: Thank you for pointing this out. PG CNDs have excellent photoluminescence performance (such as high emission quantum yield, optical stability, and narrow emission spectrum), and they can overcome many of the limitations of conventional fluorescent dyes. The common dyes for lipids are Nile Red and BODIPY. These commercial dyes possess certain limitations, making the design of new fluorescent probes for lipidic mesophases attractive. For example, Nile Red shows broad emission spectra, while BODIPY exhibits limited photostability and a small Stokes shift. [3-4] When considering multicolor imaging, these drawbacks may make them unsuitable for this application.

We have compared the optical properties of PG CNDs with Nile Red. CYAN and GREEN CNDs show narrower emission peaks than Nile Red, and they exhibit full width at half maximum (FWHM) of around 32 nm, 33 nm, and 62 nm, respectively (**Figures S1 and S2a**). Narrower FWHM results in higher spectral resolution and reduced spectral overlap with other fluorescent probes, which can be essential in bioimaging. Furthermore, Nile Red and PG CNDs show high photostability under NIR irradiation with the pulsed laser beam (**Figure S2b**). The manuscript has been supplemented.

Page 7, line 5:

Compared to the commercial lipophilic stain Nile Red (FWHM ~62 nm, Figure S2a), CYAN and GREEN CNDs exhibit narrower emission spectra. In the case of YELLOW CNDs, the FWHM value is comparable with Nile Red's value. Moreover, these yellow-emitting NPs have a significant Stokes shift (3186 cm^{-1}), which may benefit multiplex imaging.(49, 51-52) Furthermore, PG CNDs show excellent photostability under prolonged illuminations. As illustrated by the example of YELLOW CNDs in comparison with Nile Red (Figure S2b), CNDs exposed to continuous NIR-irradiation with the pulsed laser beam (λ = 740 nm, P_{laser} = 10 mW) also maintain high fluorescence intensity.

Page 17, line 1:

Additionally, selecting PG CNDs with narrow emission spectra at specified spectral ranges to stain lipidic mesophases can be crucial to optimizing spectral overlap in the simultaneous imaging of multiple fluorophores.

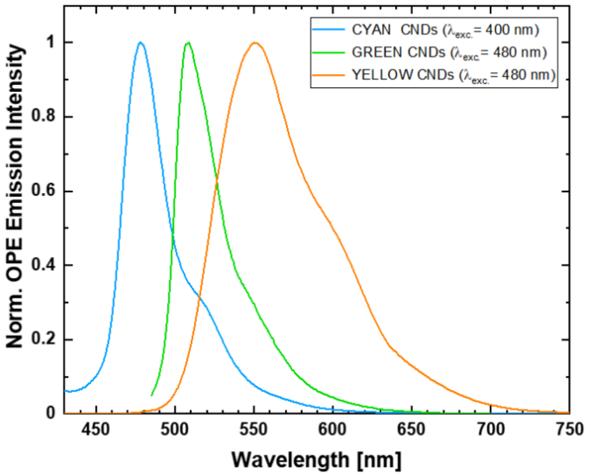

***Figure S1** Normalized one-photon excited (OPE) emission spectra of multicolor emitting PG CNDs dispersed in methanol. The samples were excited at 400 nm (CYAN CNDs) and 480 nm (GREEN and YELLOW CNDs). Materials were reproduced from Mucha et al. with permission the Royal Society of Chemistry.*[1]

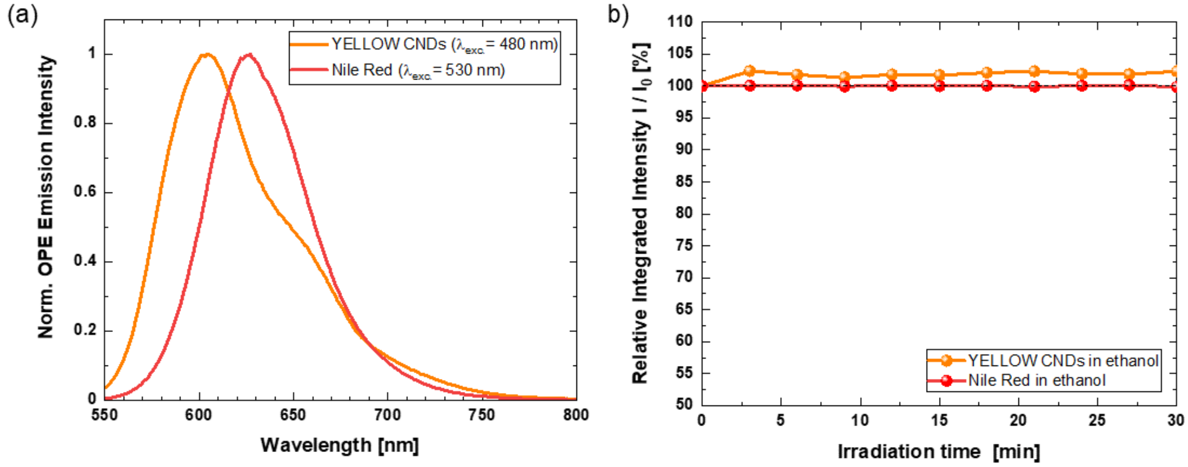

***Figure S2 (a)** OPE emission spectra of YELLOW CNDs and Nile Red dispersed in EtOH. The samples were excited at 480 nm (YELLOW CNDs) and 530 nm (Nile Red). **(b)** Photostability of YELLOW CNDs (marked in orange) and Nile Red (marked in red) in EtOH under continuous irradiation with the pulsed laser beam (λ = 740 nm, P_{laser} = 10 mW). YELLOW CNDs and Nile Red were excited at 480 and 530 nm, respectively. The black dashed line indicates 100% level.*

6. As the imaging application of CNDs is in water media. The PLQYs of these CNDs in water should be given.

Author reply: To answer this question, we focus on CYAN CNDs in 10 mM Tris Buffer (pH = 7.4). First, one-photon excited emission has been measured to be slightly blue-shifted (at 464 nm) as compared to methanol dispersion (at 478 nm). Second, we have performed an analysis of the PLQY of CYAN CNDs. The PLQY values are estimated to be ~34.1%; this value is nearly two times lower than the emission efficiency of methanol samples (~61.8%) but it is still high enough to consider CYAN CNDs as effective fluorescent markers.

Page 6, line 23:

PG CNDs are excellent fluorophores (e.g. the photoluminescence QY of CYAN CNDs is around 62% in methanol (MeOH) dispersion and 34% in Tris Buffer) and efficient two-photon absorbers (e.g. two-photon absorption merit factor reaches 0.31 GM·mol·g^{-1} at 740 nm for YELLOW CNDs; Table S1.).(49)

SI, Page S3, line 14:

The absolute fluorescence quantum yield of CYAN CNDs[1] in 10 mM Tris Buffer (pH = 7.4) was determined using a home-built experimental setup, consisting of an integrating sphere, a neutral density filter (Thorlabs), a high-sensitivity spectrometer (QE Pro, Ocean Optics), and a BDL - 375 - SMN Picosecond Laser Diode (377 nm) as an excitation source.

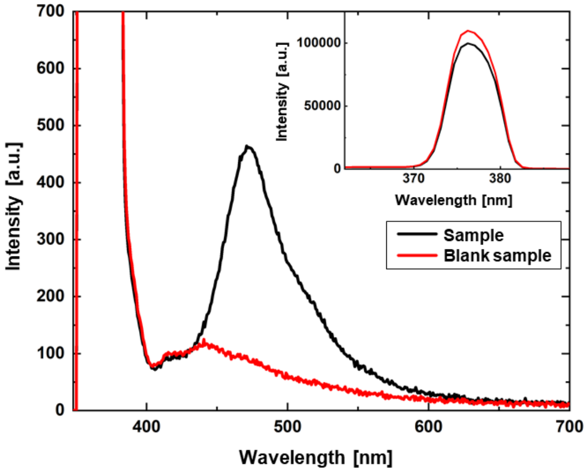

Figure S7 *The one-photon excited fluorescence spectra of CYAN CNDs in 10 mM Tris Buffer (pH = 7.4; sample) and the corresponding blank sample ($\lambda_{exc.}$ = 377 nm).*

Reviewer: 3

Recommendation:

This paper may be publishable, but major revision is needed; I would like to be invited to review any future revision.

Comments:

In this manuscript, Matczyszyn and coworkers reported labelling of MFs by biocompatible and strongly emitting multicolour phloroglucinol-derived carbon nanodots (PG CNDs) using fluorescence microscopy and spectroscopy techniques along with polarized light microscopy. The authors reported the stability and morphology of lipidic mesophases. The author claimed that PG CNDs can be a viable and simple alternative to conventional fluorescent lipid stains to image biologically relevant phospholipid-based structures. The manuscript may be publishable in JPC Letters, However, I am not convinced by the urgency of the publication. So, I am recommending a major revision for this manuscript. The authors should address the following issues before publications.

1. A similar work has been recently published by the authors (J. Phys. Chem. B 2020, 124, 11974−11979). Thus, the claims in the manuscript are not very new. The authors should explain in detail the additional advantage of taking carbon dots? The manuscript lacks new insight regarding the interaction between myelin and lipid bilayer.

Author reply: Thank you for your comments. The manuscript presents the potential use of multicolored nanomaterials for labeling the hydrophobic phase of PC-based MFs. It is the first article on this topic. In our previous article (J. Phys. Chem. B 2020, 124, 11974−11979) we showed various morphologies of MFs (straight, twisted, and oval with solid and hollow cross-sectional view). Here, we confirm the presence of characteristic shapes of MFs doped with PG CNDs (**Figure S3).** Additionally, we demonstrated using two-photon excited fluorescence microscopy for imaging MFs in three dimensions. The use of this technique also complements the previous publication. PG CNDs within PC-based MFs were efficiently excited by the absorption of nearly 2-times lower energy photons than in the one-photon excited fluorescence microscopy, essential for high-resolution, non-invasive bioimaging. Moreover using PG CNDs as fluorescent stains can provide optimization of multicolor imaging experiment since the appropriate choice of these NPs enables distinct fluorescence signals across different spectral ranges.

Page 3, line 24:

(…) there is still a lack of studies on doping hydrophobic regions of MFs with NPs and imaging lipidic mesophases with fluorescent probes offering multicolor emission upon excitation in biological windows.(31)

Page 7, line 7:

(…) these yellow-emitting NPs have a significant Stokes shift (3186 cm^{-1}), which may benefit multiplex imaging.(49, 51-52) Furthermore, PG CNDs show excellent photostability under prolonged illuminations.

Page 13, line 6:

Since CYAN CNDs were excited at a wavelength in the UV, we performed further imaging of nature-inspired structures using excitation wavelengths from the NIR-I region. For this purpose, we applied two-photon excited fluorescence microscopy (TPEFM), which overcomes many of the limitations of conventional (i.e. one-photon) techniques, allowing deeper penetration depth and better image contrast. (31, 56) Furthermore, incorporating multiphoton microscopy for lipidic mesophases aligns with the current trend of imaging nerve fibers in high-volume samples.(32, 57)

Page 17, line 1:

Additionally, selecting PG CNDs with narrow emission spectra at specified spectral ranges to stain lipidic mesophases can be crucial to optimizing spectral overlap in the simultaneous imaging of multiple fluorophores. Considering that carbon-based NPs are in line with the trend of environmentally friendly materials and the excellent performance of PG CNDs, we demonstrated an appealing alternative to commercial lipophilic fluorescent molecules for exploring biologically relevant PC-based structures.

2. It is not clear why the authors chose PG CNDs. There are a large numbers of carbon dots available in the literature which are often used for fluorescence and imaging. The PG CNDs are not exceptional. I expect that the other carbon dots with wide range of emission will exhibit a similar morphology like the PG CNDs do. The author needs to do the experiment with at least one conventional CNDs to substantiate the superiority of PG CNDs

Authors' reply: The Reviewer mentioned very interesting issues. We have chosen phloroglucinol-derived CNDs due to their excellent optical performance. As we reported in our previous work, three types of new PG CNDs were synthesized to provide intense emission signals with high color purity in different spectral regions: from cyan to yellow/orange. Their absorption and emission properties were extensively characterized in one- and two-photon regimes. We confirmed clearly that the OPE and TPE fluorescence can be tuned with the internal structure and the chemical

environment - this constitutes an exception for other CNDs which provided both OPE and TPE emission. The full characteristics of PG CNDs were included in the previous work; therefore, this manuscript could focus only on the development of their application with no need for additional characterizations (*e.g.* TPE emission or color tunability). Moreover, each type of our PG CNDs possesses nearly the same size which is comparable with the thickness of bilayers composed of phosphatidylcholines (do not exceed 5 nm).

We have supplemented the manuscript with images performed by HR-TEM (**Figure 2a and 2b**) and described the optical performance of PG CNDs in comparison to the commonly used lipid dye (Nile Red).

Our recent article demonstrated the possibility of labeling the aqueous phase of MFs with hydrophilic folic acid-based CNDs. We reference this work in the manuscript, and exemplary results are presented below. In our paper, we have confirmed that CNDs label the hydrophobic parts of the myelin figures and do not accumulate in their water cores (**Figure R7**). [2] Additionally, we compared the use of PG CNDs and the common lipophilic dye, which possesses certain limitations (such as broad emission spectra).

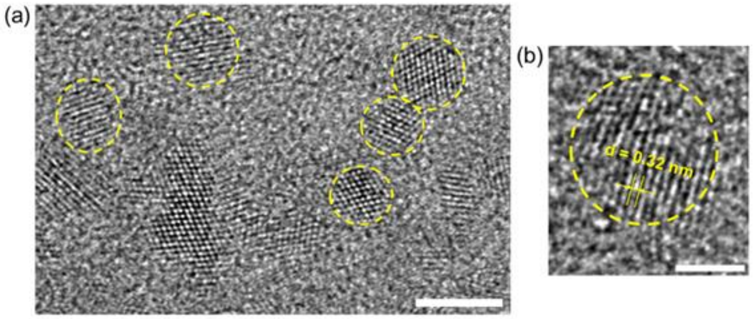

Figure 2 (a-b) *TEM images of YELLOW CNDs.*

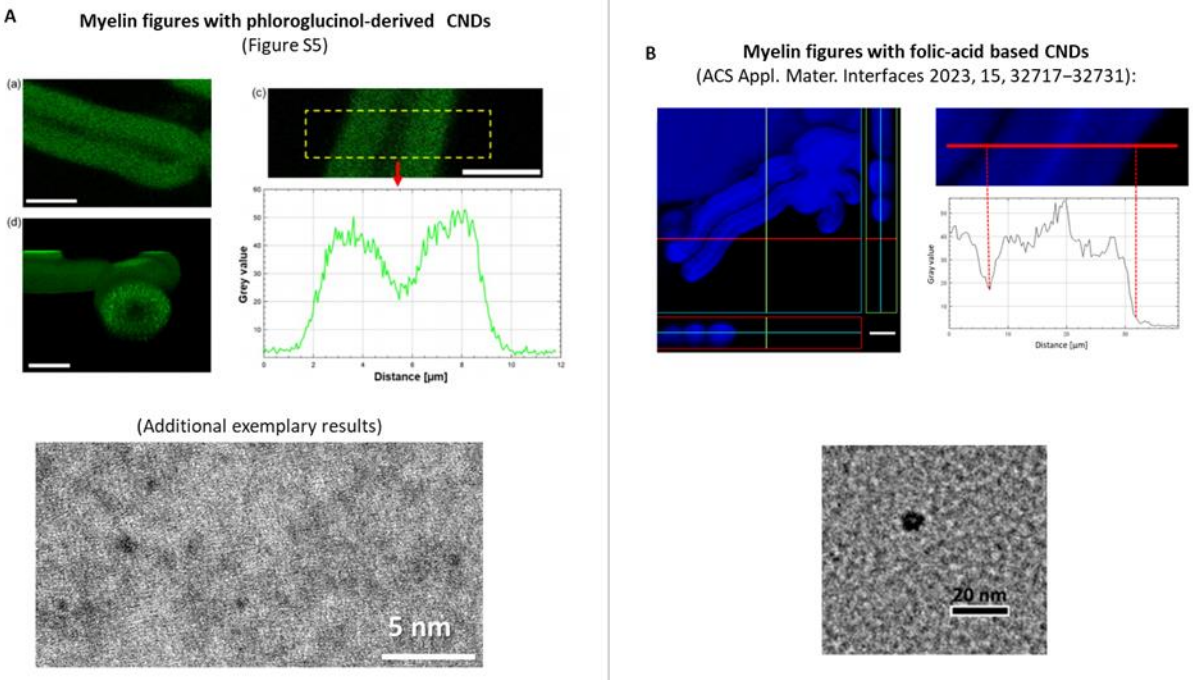

Figure R7 **(A)** The confocal fluorescence microscopy (CFM)image of DLPC-based MFs marked with GREEN CNDs taken upon excitation at 480 nm (λ_{em}: 510 - 520 nm) and the fluorescence intensity plot profile was obtained for the area marked by a yellow square. The x-axis shows the horizontal distance within the selected area, and grey values represent vertically averaged pixel intensity. The scale bars represent 5 µm. Images marked as a-c in **(A)** are presented in the **Figure S5**. TEM image of YELLOW CNDs is additional exemplary result. **(B)** CFM images of the MFs stained with folic-acid based CNDs with cross-sectional views and **a** line profile of pixel intensity taken along the red line of the CFM image shown above. Images shown in (B) are reproduced from our previous article.[2] The scale bar represents 10 µm. TEM image show folic-acid based CNDs.

Page 3, line 23:

Moreover, carbon nanodots (CNDs) have been successfully applied in staining the aqueous phase in multilamellar lipid tubes.(30) However, there is still a lack of studies on doping hydrophobic regions of MFs with NPs and imaging lipidic mesophases with fluorescent probes offering multicolor emission upon excitation in biological windows.(31)

Page 7, line 5:

Compared to the commercial lipophilic stain Nile Red (FWHM ~62 nm, Figure S2a), CYAN and GREEN CNDs exhibit narrower emission spectra. In the case of YELLOW CNDs, the FWHM value is comparable with Nile Red's value.

Page 17, line 1:

Additionally, selecting PG CNDs with narrow emission spectra at specified spectral ranges to stain lipidic mesophases can be crucial to optimizing spectral overlap in the simultaneous imaging of multiple fluorophores.

3. The author mainly worked with the zwitterionic lipid. What would be the effect of ionic lipid on the MF morphology? All the experiments were conducted in Mili-Q water, is there is any role of buffer in governing the interaction between lipid and Myelin.

Although the authors emphasized on the morphology of MF, it would be interesting to get the morphology using different lipid at different condition.

Authors' reply: The Reviewer has raised very interesting questions. Lipids with a small polar head have a molecular shape that resembles a truncated cone and facilitate the organization of membranes into inverted micelles or cubic structures. Lipids with comparable cross-sectional dimensions for both the polar head and hydrophobic region (such as PC) adopt a cylindrical shape and form lamellar phases.[5] We prepared structures made of mixture of DLPC and DMPS lipids (2:1) in an aqueous environment ($T_{m\ DLPC}$ = -2 °C , $T_{m\ DMPS}$ = 35 °C). DLPC is a zwitterionic phospholipid, while DMPS is a phospholipid with a negative charge due to the presence of serine in the polar head. As shown in **Figure R9**, the structures built from this mixture grew irregularly and twisted. The examination of phospholipid-based MFs composed of lamellar forming lipids and their mixtures with ionic lipid (such as PS) requires further exploration, particularly in the context of studying more complex models with compositions similar to the natural myelin sheath.

Moreover, we have performed observations by polarized light microscopy for PC-based MFs formed over a wide range of pH.[8] We compared the diameters of structures composed of DLPC and DMPC (saturated lipids) at different pH. We have shown that the diameter of multilamellar lipid tubes is narrower in the case of lipids with shorter carbon chains. This trend was observed across the entire temperature range (**Figure R10**). There was a negative correlation between T_m and pH, which may result from varying charges of the lipid headgroup. In addition, the size of the MF was found to be strongly dependent on the initial conditions of the isotropic phase, showing a decreasing tendency of diameter with increasing pH. Besides, the following growth process over a wide range of pH values led us to conclude that the acidic medium facilitates the elongation of MFs. The change in the growth rate of MFs can be related to the reduced interfacial tension of lipid bilayers at low pH. The correlation between pH of surroning medium and dimensions of MFs was also observed in lipidic mesophases composed of unsaturated DOPC lipid.[9]

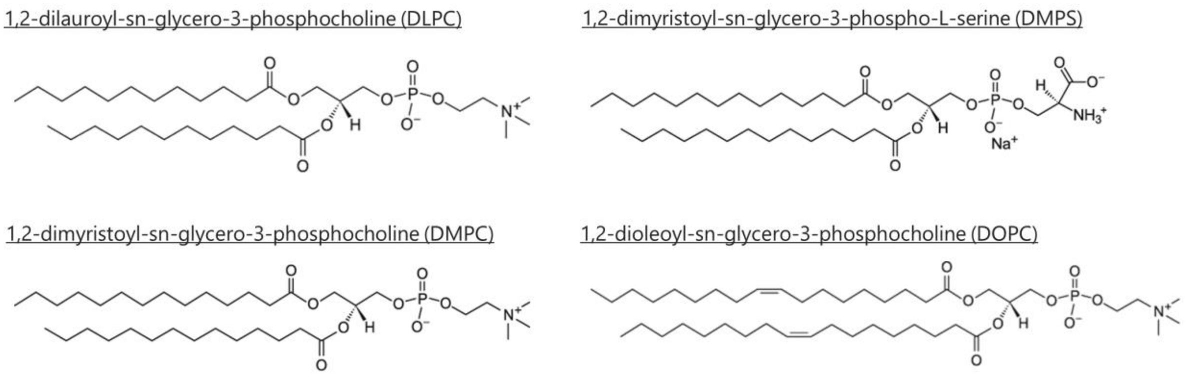

Figure R8 The structural formulas of the abovementioned phospholipids.

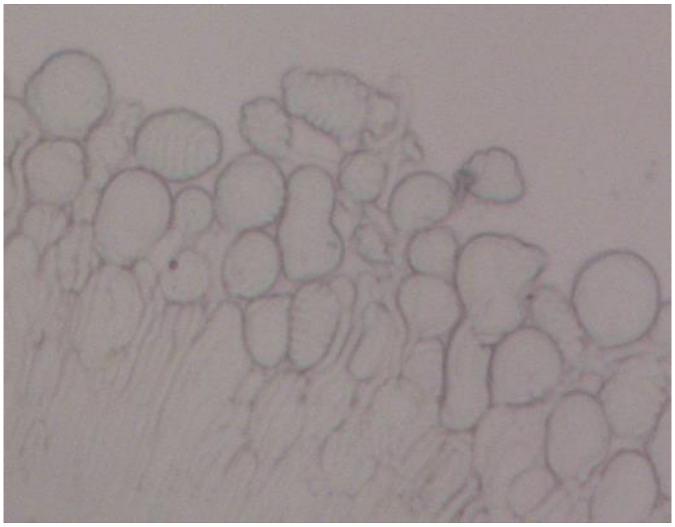

Figure R9 Bright field image of structures made of the mixture of DLPC and DLPS at 40 ° taken with objective 10x.

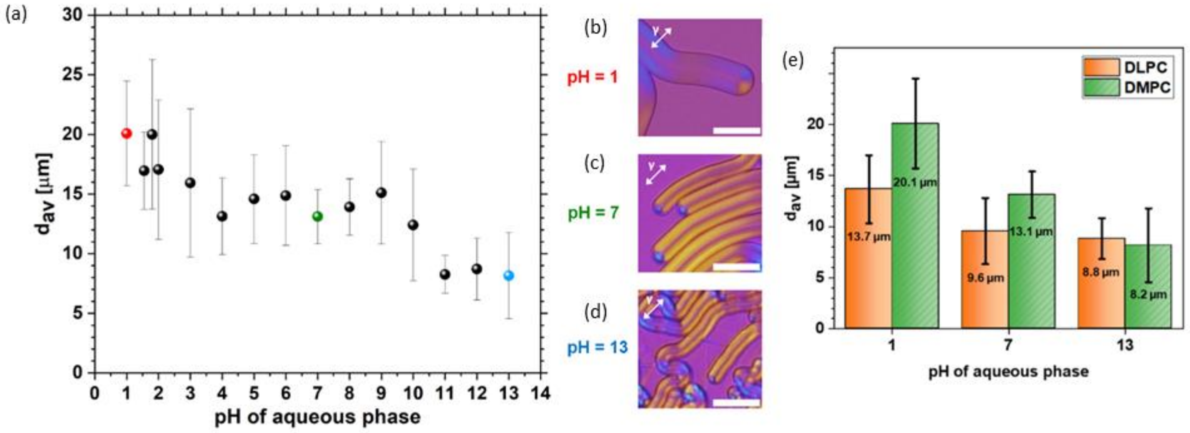

Figure R10 (a) Plot showing the average diameter of MFs vs. the pH of the aqueous phase. (b-d) Representative polarized light images of pristine MFs were performed after the addition of (d) acidic, (e) neutral, and (f) alkaline solutions. The white double arrows show the orientation of the slow axis of the retardation plate. Scale bars correspond to (d-f) 30 µm. (e) The representative average diameters of MFs composed of DLPC (orange, plain pattern) at 24 ◦C or DMPC (green, diagonal pattern) at T_m; lipids were dissolved in chloroform, dried, and then hydrated at certain temperatures by acidic, neutral and alkaline solutions. Materials were reproduced from Benkowska-Biernacka *et al*.[6]

In our manuscript, we have followed the diameters of MFs made of DMPC (stained with different concentrations of PG CNDs) formed at acidic and neutral media (Figure 2e). Results are presented in the main text and supporting information.

Page 16, line 14:

Our results indicated that mesophases containing PCs and the appropriate concentration of PG CNDs formed at a neutral pH retain the liquid crystalline properties of pristine MFs. With PLM, we demonstrated that the presence of NPs does not disturb the formation of the distinctive morphologies of MFs.

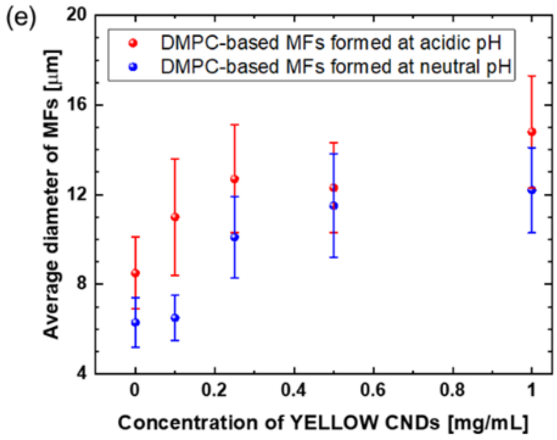

Figure 3 (e) *Graph depicting the average diameter of DMPC-based MFs formed with and without YELLOW CNDs at acidic (red dots) and neutral (blue dots) pH.*

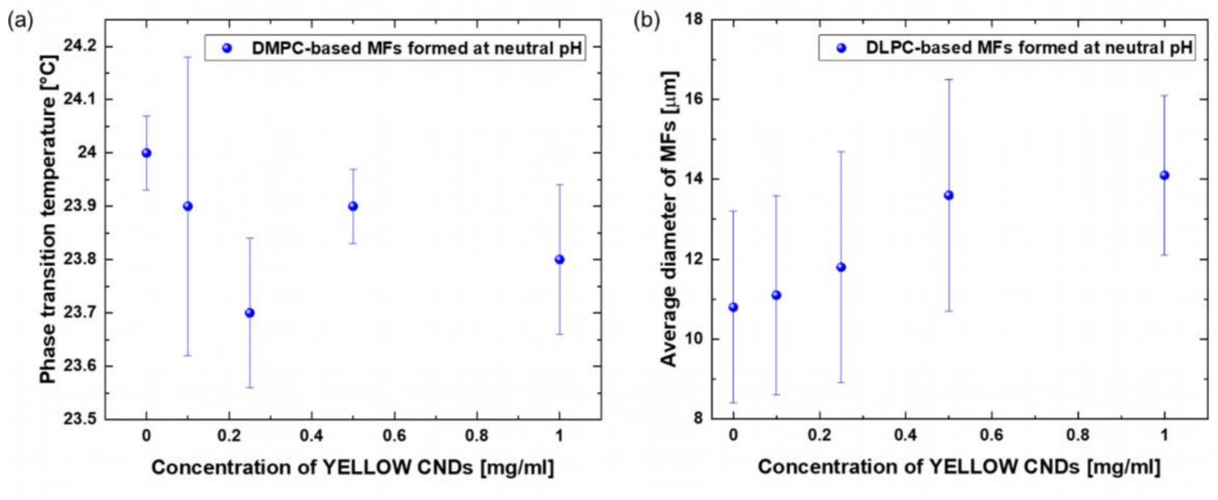

Figure S4 (a) *Scatter plot indicating the formation temperature of DMPC-based MFs with and without CNDs after hydration of a dried lipid droplet with a solution at pH = 1.* ***(b)*** *Graph depicting the average diameter of DLPC-based MFs formed with and without YELLOW CNDs at acidic (red dots) and neutral (blue dots) pH*.

4. It would be better if images of blank liposome by PG CND is shown. The exact location of PG CNDs is not well understood in the manuscript.

Authors' reply: So far, we have conducted preliminary experiments only with multilamellar vesicles (MLVs) doped with PG CNDs (**Figure R11**). In this case, no preferential alignment of the dots was observed. We have not observed CNDs localization exclusively on the outside or inside of the studied structure, which was observed for some previously described CNDs.[8] In the future, we plan to include work on liposomes (either large unilamellar or giant unilamellar vesicles) in our research. We would like to acquire a DLS instrument to optimize the parameters to obtain specific vesicles. Unfortunately, we currently do not have easy access to such device.

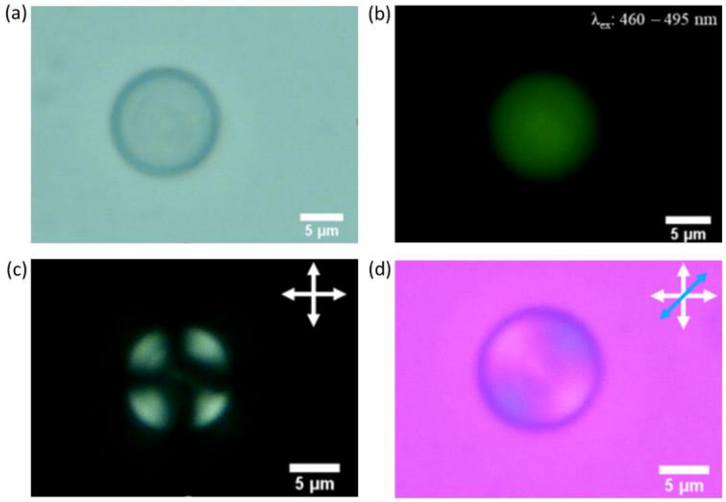

Figure R11 (a) Bright-field image and the corresponding **(b)** widefield fluorescence microscopy and **(c-d)** polarized light microscopy images of the DLPC-based liposome stained with GREEN CNDs. The arrows indicate the orientations of crossed polarizers (white double arrows) and the full-wavelength retardation plate's slow axis (blue double arrow).

In addition, studies on MFs have indicated that fluorescence from CNDs is not observed in the isotropic core of the phospholipid-based structure, but only in parts containing the stacks of lipid bilayers (e.g. in **Figure 4c and 4d**). Importantly, we showed a homogeneous distribution of fluorescence intensity along the entire length of the MFs (e.g. in **Figure 1f**).

Page 8, line 4:

As shown in Figure 1f, the one-photon excited (OPE) emission along the lipid tubes stained with CYAN CNDs was intense and uniformly distributed within the structures that emerged from the PC/water interface ($\lambda_{exc.}$ = 360 nm). Also, there was no accumulation of nanostructures near the roots of MFs.

Page 12, line 12:

Each of the 3D scans confirmed the homogeneous distribution of PG CNDs in the lipid bilayers surrounding the water core. There was no difference in the emission intensity along areas of the lamellar mesophase wrapped around the non-fluorescent channel.

Page 16, line 12:

Moreover, regions of noticeably higher or lower intensity were not identified within the MF, indicating a lack of preferential distribution of PG CNDs in the specific areas of the sample.

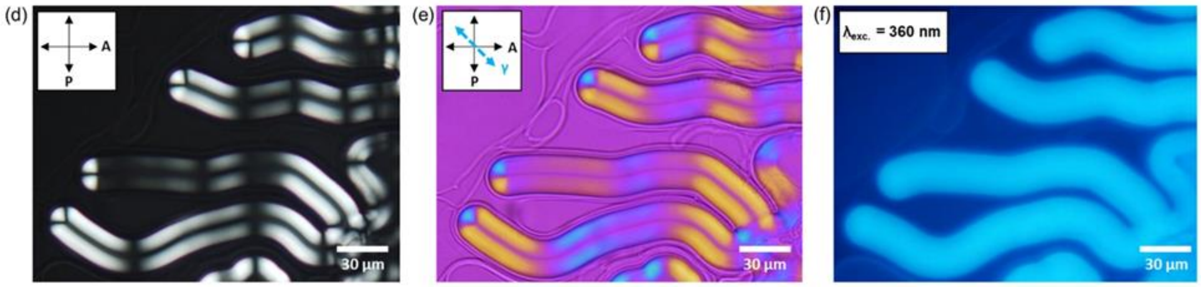

Figure 1 (d-e) *DMPC-based MFs doped with CYAN CNDs formed at the edge of the lipid droplet. The samples imaged (**d**) between crossed polarizers and **(e)** crossed polarizers with an additionally inserted full-wave retardation plate. (**f**) Fluorescence microscopy images of corresponding regions of the sample ($\lambda_{exc.}$ = 360 nm). Scale bars represent 30 µm.*

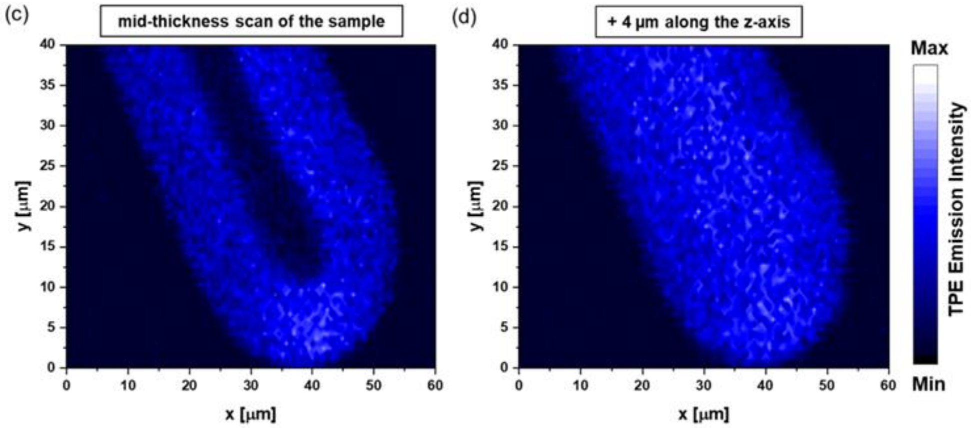

Figure 4 (c-d) *TPE fluorescence intensity maps of the same DLPC-based MF taken at mid-height of the sample and 4 µm higher. Both scans are depicted using the same intensity scale. The sample was excited at 850 nm.*

5. In figure 5, we observed that there is a redshift in the emission of CNDs in presence of lipid. The author mentioned that hydrogen bonding is responsible for the observed shift. I was curious whether this kind of CND undergo aggregation in presence of lipid molecules. The emission may come from the aggregated form. The author needs to address this issue.

Authors' reply: Thank you for your comment. The aggregation of PG CNDs in the presence of phospholipids could be investigated using dynamic light scattering (DLS). We would perform a series of experiments with different lipid concentrations. However, we do not have access to this technique right now. In the future, we would like to gain insights into the aggregation behavior of nanoparticles using DLS and, preferably, fluorescence correlation spectroscopy (a method with single-molecule sensitivity).

6. The author claimed that the diameters of the DMPC-based MFs were enhanced at acidic and neutral pH. They attributed to this protonation of phosphate group. What would be impact on the diameter of the tube if a lipid of lower headgroup is used (with higher phase transition temperature)?

Authors' reply: Thank you for this question. As we have mentioned in a previous response (the third question from the third Reviewer), lipids with a small polar head exhibit the molecular shape of a truncated cone, facilitating the organization of membranes into inverted micelles or cubic structures. We have investigated structures composed of a mixture of DLPC and DMPS lipids (2:1) in an aqueous environment (T_m for DLPC is of -2°C and 35°C for DMPS). DLPC is a zwitterionic phospholipid, while DMPS carries a negative charge due to the presence of serine in its polar head. As depicted in **Figure R12**, structures formed from this mixture exhibited irregular morphologies, different from pristine PC-based MFs. Further exploration is needed to examine MFs composed of lamellar-forming lipids and their mixtures with ionic lipids (such as phosphatidylserine), especially to develop more complex models with a composition resembling the natural myelin sheath.

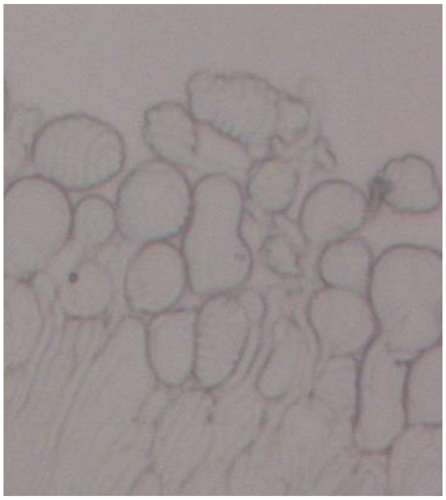

Figure R12 Bright field image of structures composed of the mixture of DLPC and DLPS at 40 °. The image shows the spontaneous growth of structures facing the aqueous phasefrom the lipid-water interface.

7. The authors need to confirm whether CDN do not perturb the morphology of the lipid bilayer. The location of CDN is very important. It needs to be confirmed if CDN undergo homogeneous distribution in the lipid bilayer surface.

Authors' reply: Thank you for bringing this matter to our attention. We performed studies on MFs formed in the presence of various amounts of PG CNDs. We followed in detail the influence of

the addition of YELLOW CNDs on the diameter of elongated multilamellar structures. Based on the results presented in **Figure 2e**, we mainly used PG CNDs dispersed in EtOH solution at a concentration of 0.1 mg/mL. In this case, the difference in diameter of MFs was around 3% compared to pristine MFs formed under the same conditions. We did not observe the accumulation of nanoparticles in any specific area of MFs (e.g. on the top or its outer edges), even if the initial concentration of NPs was 1 mg/mL (**Figure 2c)**. According to the analysis of HR-TEM images (**Figure 2a** and results from the previous publication by Mucha et al.[1]), the diameter of these PG CNDs is around 4 nm, which is similar to the average thickness of fully hydrated lipid bilayers (for instance 3.67 nm at 25 ◦C for DMPC[9]).

We reported the formation of various characteristic structural morphologies of multilamellar tubes stained with PG CNDs, including straight, oval and looped (**Figure S3**). These shapes are characteristics of PC-based MFs.[10] What is more, using a polarized light microscope with a full-wave retardation plate, we determined the orientation of lipids within the lamellar phase (i.e. walls of MFs). The aliphatic tails of the phosphatidylcholines within MFs marked with PG CNDs are oriented to be parallel and perpendicular to the water core, which is also typical for MFs. Even though the addition of PG CNDs increases the diameter of PC-based MFs, characteristic structures are observed in samples with NPs at each of the investigated initial concentrations.

Moreover, we have confirmed that CNDs label the hydrophobic parts of the MFs and do not accumulate in their water cores (as shown in, for instance, **Figure 4 c-d**). These results can be compared with findings obtained for phospholipid-based MFs marked with hydrophilic folic acid-based CNDs, which stained the inner channel running along the MFs and water layers alternating phospholipid bilayers (shown in an answer to the second question asked by the third Reviewer).[2] Homogeneous distribution of fluorescence intensity was observed over the entire length of the multilamellar tubes stained with PG CNDs. No higher emission signal was detected, even in the bent regions of oval structures (e.g., **Figure 3b**).

We have also supplemented the manuscript with information on the presence of non-polar moieties in PG CNDs.

Page 12, line 12:

Each of the 3D scans confirmed the homogeneous distribution of PG CNDs in the lipid bilayers surrounding the water core. There was no difference in the emission intensity along areas of the lamellar mesophase wrapped around the non-fluorescent channel.

Page 6, line 16:

Furthermore, PG CNDs consist of polar (e.g. hydroxyl groups) and non-polar (methyl and methylene moieties) structural units. Their extensive structural characterization unravelled the predominant role of non-polar carbogenic domains. (49)

Page 7, line 23:

Figure 1e shows that the characteristic organization of lipid molecules along the pristine MFs is also observed in mesophases doped with PG CNDs. (15, 53) The amphiphilic molecules are self-organized over the length of the MFs in a way that they are arranged into a concentric lamellar structure. (…) As shown in Figure 1f, the one-photon excited (OPE) emission along the lipid tubes stained with CYAN CNDs was intense and uniformly distributed within the structure that emerged from the PC/water interface ($\lambda_{exc.}$ = 360 nm). Also, there was no accumulation of nanostructures near the roots of MFs.

Page 6, line 12:

Based on high-resolution transmission electron microscopy analysis, PG CNDs have irregular shapes and average diameters of ~ 4 nm (exemplary results are in Figure 2a).

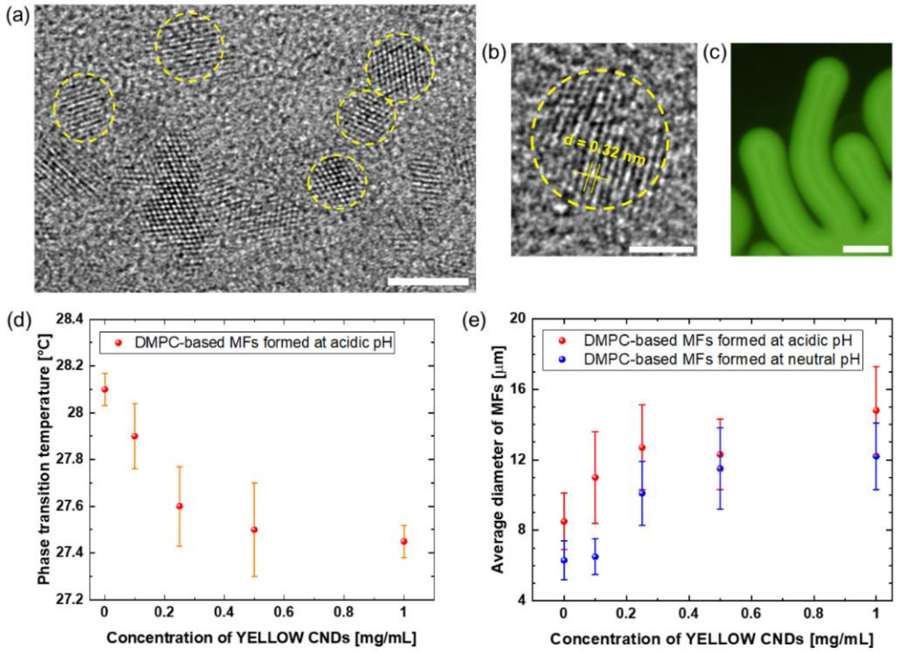

***Figure 4 (a-b)** TEM images of YELLOW CNDs. **(c)** Widefield fluorescence microscopy image of MFs with YELLOW CNDs (at initial nanoparticle concentration of 1 mg/mL) formed at acidic pH ($\lambda_{exc.}$: 460 – 495 nm). Scale bars are **(a)** 5 nm, **(b)** 2 nm and **(c)** 20 µm. **(d)** Scatter plot indicating the temperature at which DMPC-based MFs start to form with and without CNDs after hydration of a dried lipid droplet with a solution at pH = 1. **(e)** Graph depicting the average diameter of DMPC-based MFs formed with and without YELLOW CNDs at acidic (red dots) and neutral (blue dots) pH.*

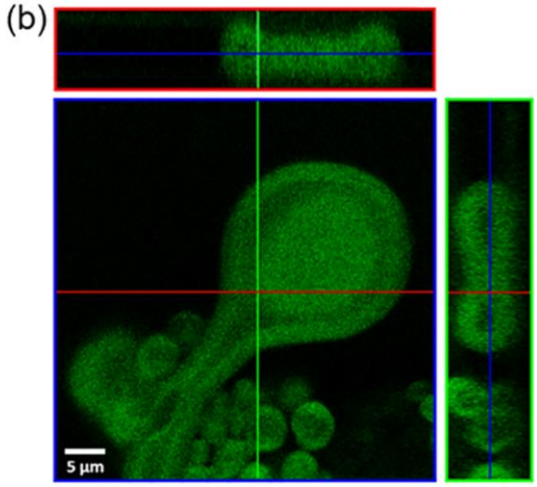

Figure 5 Fluorescence confocal images of MFs doped with GREEN YELLOW CNDs taken upon excitation at 480 nm. The emission signal was collected at wavelength ranges 510 – 520 nm. Scale bars are 5 µm.

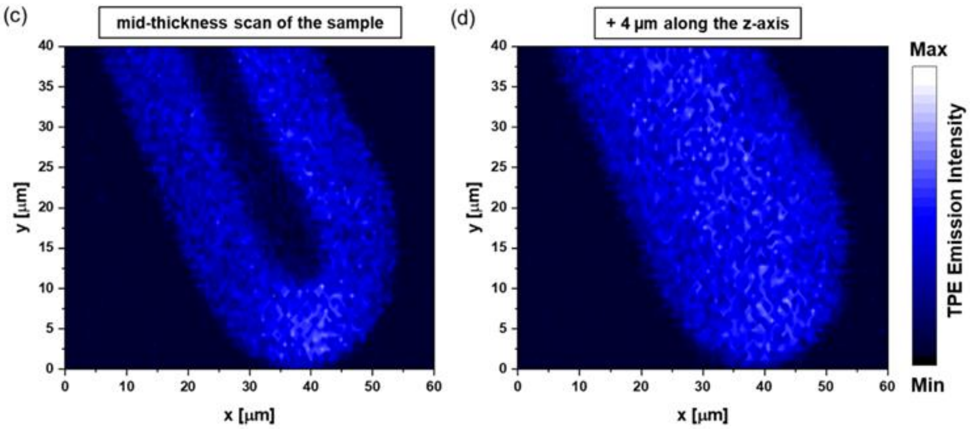

Figure 4 (c-d) TPE fluorescence intensity maps of the same DLPC-based MF taken at mid-height of the sample and 4 µm higher. Both scans are depicted using the same intensity scale. The sample was excited at 850 nm.

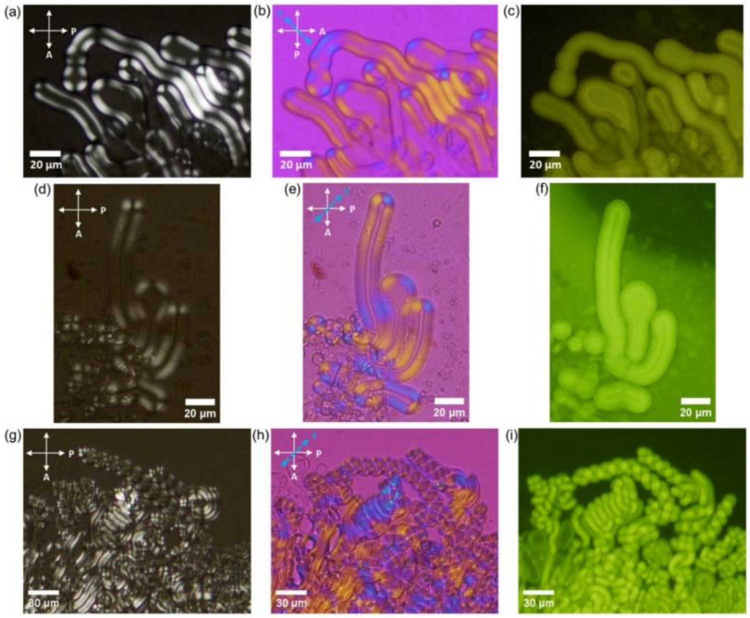

__Figure S1__ Imaging of different forms of MFs doped with (a-c) GREEN and (d-i) YELLOW CNDs using polarized light (left and center columns) and widefield fluorescence microscopy under excitation in the range of 460 – 495 nm (right column). The orientations of two polarizers ('A' and 'P') are indicated by white double-sided arrows and the direction of the slow axis of the retardation plate (γ) is marked as a blue double arrow. Scale bars are __(a-f)__ 20 µm and __(g-i)__ 30 µm.

8. A new insight may be given regarding the interaction of myelin and lipid bilayer interaction.

Authors' reply: Thank you for your comment. Lipidic MFs are elongated 3D forms that consist of hundreds of concentrically wrapped lipid bilayers interspaced with layers of water. These structures are formed above the main phase transition temperature (T_m), where lipids are in the liquid crystalline phase. Under these conditions, the hydrocarbon chains are disordered as a result of the trans-gauche isomerization of some of the C–C bonds.[8] As mentioned in the answer to the first question of the third Reviewer, we demonstrated three-dimensional imaging of the hydrophobic part of MFs using two-photon excited fluorescence microscopy (2PEFM). Among other advantages, 2PEFM offers deeper sample penetration and less photodamage. This imaging technique could be applied here as we stained MFs with strongly emitting CNDs, which can be efficiently excited in the NIR-I region. These findings complement our earlier publication on the morphology of PC-MFs and staining isotopic phases in multilamellar lipidic mesophases with blue-emitting FA CNDs.[2] Moreover, using multicolor PG CNDs as lipophilic probes paves the way for improving experiments in which simultaneous detection of multiple fluorophores is crucial.

Page 3, line 24:

(…) there is still a lack of studies on doping hydrophobic regions of MFs with NPs and imaging lipidic mesophases with fluorescent probes offering multicolor emission upon excitation in biological windows.(31)

Page 16 , lines 19:

Further microscopic and spectroscopic studies proved that multicolored PG CNDs in a lipidic matrix can be excited in linear and nonlinear regimes. The presented findings showed that PG CNDs can serve as fluorescent probes to efficiently image various shapes of PC-based multilamellar microstructures. Using the non-invasive and high-resolution technique, such as TPEFM, to detect PG CNDs-doped lipidic mesophases in 3D may have significant implications in bioimaging, as even subtle changes in the lipid-rich myelin sheath can impact axonal conduction. Additionally, selecting PG CNDs with narrow emission spectra at specified spectral ranges to stain lipidic mesophases can be crucial to optimizing spectral overlap in the simultaneous imaging of multiple fluorophores.

References:

1. Mucha, S. G.; Firlej, L.; Formalik, F.; Bantignies, J.-L.; Anglaret, E.; Samoć, M.; Matczyszyn, K., Revealing two chemical strategies to tune bright one- and two-photon excited fluorescence of carbon nanodots. *J. Mater. Chem. C* **2024,** *12* (6), 2117-2133.

2. Benkowska-Biernacka, D.; Mucha, S. G.; Firlej, L.; Formalik, F.; Bantignies, J.-L.; Anglaret, E.; Samoć, M.; Matczyszyn, K., Strongly Emitting Folic Acid-Derived Carbon Nanodots for One- and Two-Photon Imaging of Lyotropic Myelin Figures. *ACS Appl. Mater. Interfaces* **2023**.

3. Fam, T. K.; Klymchenko, A. S.; Collot, M. Recent Advances in Fluorescent Probes for Lipid Droplets *Materials* [Online], 2018.

4. Rumin, J.; Bonnefond, H.; Saint-Jean, B.; Rouxel, C.; Sciandra, A.; Bernard, O.; Cadoret, J.-P.; Bougaran, G., The use of fluorescent Nile red and BODIPY for lipid measurement in microalgae. *Biotechnology for Biofuels* **2015,** *8* (1), 42.

5. Jouhet, J., Importance of the hexagonal lipid phase in biological membrane organization. *Frontiers in Plant Science* **2013,** *4*.

6. Benkowska-Biernacka, D.; Smalyukh, I. I.; Matczyszyn, K., Phase behavior of phospholipid-based myelin figures influenced by pH. *J. Mol. Liq.* **2023,** *391*, 123365.

7. Allah Panahi, M.; Tahmasebi, Z.; Abbasian, V.; Amiri, M.; Moradi, A.-R., Role of pH level on the morphology and growth rate of myelin figures. *Biomedical Optics Express* **2020,** *11* (10), 5565-5574.

8. Kanwa, N.; M, K.; Chakraborty, A., Discriminatory Interaction Behavior of Lipid Vesicles toward Diversely Emissive Carbon Dots Synthesized from Ortho, Meta, and Para Isomeric Carbon Precursors. *Langmuir* **2020,** *36* (35), 10628-10637.

9. Karjiban, R. A.; Shaari, N. S.; Gunasakaran, U. V.; Basri, M., A Coarse-Grained Molecular Dynamics Study of DLPC, DMPC, DPPC, and DSPC Mixtures in Aqueous Solution. *Journal of Chemistry* **2013,** *2013*, 1-6.
10. Benkowska-Biernacka, D.; Smalyukh, I. I.; Matczyszyn, K., Morphology of Lyotropic Myelin Figures Stained with a Fluorescent Dye. *J. Phys. Chem. B.* **2020,** *124* (52), 11974-11979.

jz-2024-00788e.R2

Name: Peer Review Information for "Three-Dimensional Imaging of Bioinspired Lipidic Mesophases Using Multicolored Light-Emitting Carbon Nanodots."

Second Round of Reviewer Comments

Reviewer: 2

Comments to the Author

The authors have answered my questions well. I recommend publication in JPCL.

Reviewer: 3

Comments to the Author

I am happy with the replies made by the authors. I recommend the manuscript for publication.

Author's Response to Peer Review Comments:

Dear Editor,

thank you and the anonymous reviewers for the favorable decision considering our manuscript.

Please find enclosed to this submission corrected references both in the main manuscript as well as in the SI.

Best regards,

Katarzyna Matczyszyn
